# ONE-seq: epitranscriptome and gene-specific profiling of NAD-capped RNA

**DOI:** 10.1093/nar/gkac1136

**Published:** 2022-12-07

**Authors:** Kongyan Niu, Jinyang Zhang, Shuwen Ge, Dean Li, Kunfeng Sun, Yingnan You, Jiaqian Qiu, Kun Wang, Xueting Wang, Rui Liu, Yandong Liu, Bing Li, Zheng-Jiang Zhu, Lefeng Qu, Hong Jiang, Nan Liu

**Affiliations:** Interdisciplinary Research Center on Biology and Chemistry, Shanghai Institute of Organic Chemistry, Chinese Academy of Sciences, 100 Hai Ke Rd., Pudong, Shanghai 201210, China; University of Chinese Academy of Sciences, Beijing 100049, China; Interdisciplinary Research Center on Biology and Chemistry, Shanghai Institute of Organic Chemistry, Chinese Academy of Sciences, 100 Hai Ke Rd., Pudong, Shanghai 201210, China; University of Chinese Academy of Sciences, Beijing 100049, China; Interdisciplinary Research Center on Biology and Chemistry, Shanghai Institute of Organic Chemistry, Chinese Academy of Sciences, 100 Hai Ke Rd., Pudong, Shanghai 201210, China; University of Chinese Academy of Sciences, Beijing 100049, China; Interdisciplinary Research Center on Biology and Chemistry, Shanghai Institute of Organic Chemistry, Chinese Academy of Sciences, 100 Hai Ke Rd., Pudong, Shanghai 201210, China; University of Chinese Academy of Sciences, Beijing 100049, China; Interdisciplinary Research Center on Biology and Chemistry, Shanghai Institute of Organic Chemistry, Chinese Academy of Sciences, 100 Hai Ke Rd., Pudong, Shanghai 201210, China; University of Chinese Academy of Sciences, Beijing 100049, China; Interdisciplinary Research Center on Biology and Chemistry, Shanghai Institute of Organic Chemistry, Chinese Academy of Sciences, 100 Hai Ke Rd., Pudong, Shanghai 201210, China; University of Chinese Academy of Sciences, Beijing 100049, China; Interdisciplinary Research Center on Biology and Chemistry, Shanghai Institute of Organic Chemistry, Chinese Academy of Sciences, 100 Hai Ke Rd., Pudong, Shanghai 201210, China; University of Chinese Academy of Sciences, Beijing 100049, China; Interdisciplinary Research Center on Biology and Chemistry, Shanghai Institute of Organic Chemistry, Chinese Academy of Sciences, 100 Hai Ke Rd., Pudong, Shanghai 201210, China; University of Chinese Academy of Sciences, Beijing 100049, China; Interdisciplinary Research Center on Biology and Chemistry, Shanghai Institute of Organic Chemistry, Chinese Academy of Sciences, 100 Hai Ke Rd., Pudong, Shanghai 201210, China; University of Chinese Academy of Sciences, Beijing 100049, China; Singlera Genomics, 500 Fu Rong Hua Rd., Pudong, Shanghai 201204, China; Department of Vascular and Endovascular Surgery, Chang Zheng Hospital, Naval Medical University, Shanghai 200003, China; IH Bluex Technology, 58 Xiang Cheng Road, Shanghai 200122, China; Interdisciplinary Research Center on Biology and Chemistry, Shanghai Institute of Organic Chemistry, Chinese Academy of Sciences, 100 Hai Ke Rd., Pudong, Shanghai 201210, China; Shanghai Key Laboratory of Aging Studies, 100 Hai Ke Rd., Pudong, Shanghai 201210, China; Department of Vascular and Endovascular Surgery, Chang Zheng Hospital, Naval Medical University, Shanghai 200003, China; Interdisciplinary Research Center on Biology and Chemistry, Shanghai Institute of Organic Chemistry, Chinese Academy of Sciences, 100 Hai Ke Rd., Pudong, Shanghai 201210, China; Interdisciplinary Research Center on Biology and Chemistry, Shanghai Institute of Organic Chemistry, Chinese Academy of Sciences, 100 Hai Ke Rd., Pudong, Shanghai 201210, China; Shanghai Key Laboratory of Aging Studies, 100 Hai Ke Rd., Pudong, Shanghai 201210, China

## Abstract

The hub metabolite, nicotinamide adenine dinucleotide (NAD), can be used as an initiating nucleotide in RNA synthesis to result in NAD-capped RNAs (NAD-RNA). Since NAD has been heightened as one of the most essential modulators in aging and various age-related diseases, its attachment to RNA might indicate a yet-to-be discovered mechanism that impacts adult life-course. However, the unknown identity of NAD-linked RNAs in adult and aging tissues has hindered functional studies. Here, we introduce ONE-seq method to identify the RNA transcripts that contain NAD cap. ONE-seq has been optimized to use only one-step chemo-enzymatic biotinylation, followed by streptavidin capture and the nudix phosphohydrolase NudC-catalyzed elution, to specifically recover NAD-capped RNAs for epitranscriptome and gene-specific analyses. Using ONE-seq, we discover more than a thousand of previously unknown NAD-RNAs in the mouse liver and reveal epitranscriptome-wide dynamics of NAD-RNAs with age. ONE-seq empowers the identification of NAD-capped RNAs that are responsive to distinct physiological states, facilitating functional investigation into this modification.

## INTRODUCTION

In eukaryotes, 5′,5′-triphosphate-linked 7-methylguanosine (m^7^G) is the predominant 5′-end cap structure of RNA (m^7^Gppp-RNA or m^7^G-RNA), essential for RNA stability, polyadenylation, splicing, localization, and translation (1–3). Recently, NAD, the adenine nucleotide containing metabolite, emerged as a non-canonical initiating nucleotide (NCIN) incorporating at the 5′-terminus of RNA to result in NAD-capped RNAs (NAD-RNA), which are estimated to make up more than 0.6% and 1.3% of the mRNA transcripts expressed in the entire transcriptome from mouse liver and kidney, respectively (4,5). NAD capping may define a yet-to-be understood epitranscriptomic mechanism.

Mounting evidence associates aging with transcriptional and metabolic alterations (6–8), yet how these processes are integrated into the regulation of aging is only beginning to be revealed. NAD is the hub metabolite and redox regent for cells, involving in a wide range of biological processes (9). In rodents and humans, studies have revealed that the NAD level declines with age in critical organs including liver (10). Boosting NAD biosynthesis, on the other hand, extends lifespan in yeast (11), worms (12), flies (13) and mice (14). Given the dynamics of NAD and gene expression over the course of adult lifespan, it is important to better understand how NAD-RNAs are modulated with age and its consequent impact on the progression of aging. Knowing the identity of NAD-modified RNAs is prerequisite for functional studies. However, investigating functional insight of NAD-RNAs has been largely limited by the analytical methods available.

The currently reported NAD-RNA identification methods involve the use of multiple chemo-enzymatic reactions (15). NAD captureSeq was initially reported, utilizing an enzymatic reaction by adenosine diphosphate ribosyl-cyclase (ADPRC) and copper-catalyzed click chemistry to biotinylate NAD-RNAs for affinity binding (15). Enriched RNAs were used for high-throughput sequencing. However, the presence of copper ions degrades RNA, in congruence with the observation that most NAD-RNAs revealed by this method are 3′-truncated (15). To preserve RNA integrity, SPAAC-NAD-seq was recently developed in *Arabidopsis*, employing the strain-promoted azide alkyne cycloaddition (SPAAC) (16). In SPAAC-NAD-seq, ADPRC first catalyzed the reaction between transcripts and azide resulting in azide-functionalized substrate. Azide-functionalized substrate can be biotin-conjugated via copper-free click chemistry (16). As a result, SPAAC reaction can preserve full-length RNA transcripts. However, ADPRC has been noted to catalyze the reaction with m^7^G-RNAs (16). As such, SPAAC-NAD-seq requires antibody-based pre-treatment to deplete m^7^G-RNA from purified mRNA to mitigate non-specific enrichment of m^7^G-capped RNAs, thus demanding additional procedures and high RNA input (16). Given these limitations, currently available methods cannot be easily applied for gene-specific assessment of RNAs that contain NAD cap.

In the present study, we demonstrate that a one-step chemo-enzymatic reaction, when combined with NudC-catalyzed elution, enables the identification of NAD-RNAs directly from total RNA. By boronate affinity, we devise an ADPRC-independent strategy to validate newly identified NAD-RNAs. We proceed to explore the utility of ONE-seq method for epitranscriptome and gene-specific assessment, revealing previously unknown NAD-modified RNAs from adult mouse livers.

## MATERIALS AND METHODS

### Ethics Statement

All animal procedures have been reviewed and approved by the Institutional Animal Care and Use Committee at the Chinese Academy of Sciences and were in accordance with the Guide for the Care and Use of Laboratory Animals of the Chinese Academy of Sciences. All efforts were made to minimize the suffering of the animals.

### Animal experimentation

C57BL/6 mice were housed in an environmentally controlled room under a 12:12 h light/dark cycle at 23°C and were fed with commercial mouse chow food and water ad libitum. To examine the efficacy of NudC-catalyzed NAD-RNA elution, livers were dissected from 12-month old C57BL/6 mice. To profile NAD-RNA epitranscriptomes, livers were dissected from 2- and 18-month old C57BL/6 mice. Dissected livers were immediately frozen in liquid nitrogen.

### HEEB reaction with NAD

NAD (1 mM, Macklin, catalog: N814818) was reacted with HEEB (*N*-[2-(2-hydroxyethoxy) ethyl]-biotinamide, CAS: 717119-80-7, 5 mM, Amatek scientific, catalog: B-1328) under the catalysis of ADPRC (25 μg/ml, Sigma-Aldrich, catalog: A9106) in 100 μl of ADPRC reaction buffer (50 mM Na-HEPES pH 7.0, 5 mM MgCl_2_) (17) at 37°C for 1 h. The reaction mixture was terminated by heating at 95°C for 5 min and centrifugated at 20 000 *g* for 10 min at 4°C. The supernatant was analyzed by HPLC and LC–MS.

### HPLC and LC–MS analysis

Samples were analyzed by an Agilent Technologies 1260 Infinity with a C18 column (Phenomenex Kinetex, 5 μm EVO C18, 100 Å, 150 × 4.6 mm) monitoring at 260 nm. The mobile phase was composed of (A) 100 mM NH_4_HCO_3_ in water and (B) acetonitrile at a flow rate of 0.4 ml/min. The injection volume was 2 μl. The gradient was as follows: 0 min 0% B, 2 min 0% B, 4 min 20% B, 17 min 40% B, 19 min 100% B, 24 min 100% B, and 30 min 0% B. The retention time of NAD and ADPR-Biotin (product) was 9.393 min and 9.929 min, respectively. The corresponding compounds were purified from HPLC and then analyzed by LC–MS by Agilent Technologies 6120 Quadrupole with a C18 column (Agilent Poroshell, 120 EC-C18, 2.7 μm, 3.0 × 50 mm). The mobile phase was composed of (A) 0.1% formic acid in water and (B) acetonitrile at a flow rate of 0.4 ml/min. The injection volume was 5 μl. The gradient was as follows: 0 min 10% B, 4 min 100% B, and 7 min 100% B.

### In vitro transcription of NAD-RNA, and m^7^Gppp-RNA

To prepare short RNA of 38 nucleotides (nt), 10 μM template oligo DNA (template sequence: 5′-GATCAC**TAATACGACTCACTATT**ACTGTGTCGTCGTCGTCTGCTGTCTCTCTCTCGCGGGC-3′; boldface letters denote the sequence of T7 class II promotor (ϕ2.5)) and (anti-sense: 5′-GCCCGCGAGAGAGAGACAGCAGACGACGACGACACAGTAATAGTGAGTCGTATTAGTGATC-3′) (18) were combined in 50 μl of 1 × Cut Smart buffer (New England Biolabs, catalog: B7204S) and annealed by heating to 95°C for 5 min. The annealing reaction was by cooling down to 4°C at a ramp rate of 0.1°C/s in a thermocycler (Eppendorf, Germany). To prepare spike-in RNA of 106 nt, 88 nt oligo nucleotide without adenine was synthesized (Genewiz) and annealed to make double-stranded DNA template. Primer extension was used to increase the length of the template to encode 106 nt RNA (template sequence: 5′-GATCAC**TAATACGACTCACTATT**ACTGTGTCGTCGTCGTCTGCTGTCTCTCTCTCGCGGGCGTGGGCGTGCGCGCTGCCGCCTGCGGCTCGCTGGCCCTGTCGTTCTTCTGCTCCTCCGGCTTGCTGCC-3′; boldface letters denote the sequence of T7 class II promotor (ϕ2.5)) and (anti-sense: 5′-GGCAGCAAGCCGGAGGAGCAGAAGAACGACAGGGCCAGCGAGCCGCAGGCGGCAGCGCGCACGCCCACGCCCGCGAGAGAGAGACAGCAGACGACGACGACACAGTAATAGTGAGTCGTATTAGTGATC-3′). To prepare spike-in ppp-RNA of 62 nt, oligonucleotide was synthesized (Genewiz) and annealed to make double-stranded DNA template (template sequence: 5′-**TAATACGACTCACTATT**AGGGAAGTGCTACCACAACTTTAGCCATAATGTCACTTCTGCCGCGGGCATGCGGCCAGCCA-3′; boldface letters denote the sequence of T7 class II promotor (ϕ2.5)) and (anti-sense: 5′-TGGCTGGCCGCATGCCCGCGGCAGAAGTGACATTATGGCTAAAGTTGTGGTAGCACTTCCCTAATAGTGAGTCGTATTA-3′). To prepare spike-in NAD-RNA (500 nt) and m^7^Gppp-RNA (500 nt) with identical sequence, oligonucleotide without adenine was synthesized (Genewiz) and were subjected to polyadenylation for poly(A) tails elongation (template sequence: 5′-**TAATACGACTCACTATT**ATGGTGTGCTTGGGCGTGGTGCTGTTCTCCGGGGTGGTGCCCTTCCTGGTCGTGCTGGTCGGCGTCGTTTTCGGCCTCTTGTTCTGCGTGTCCGGCGTGGGCGTGGGCGTTGCCTCCTTCGGCTTGCTGTCCCTGTTGTTCTTCTGCTCCTCCGGCTTGCTGCCCGTGCCCTGGCCCTCCCTCGTGTCCTCCCTGTCCTTCGGCGTGCTGTGCTTCTGCCGCTTCCCCGTCCTCTTGTTGCTGCTCGTCTTCTTCTTGTCCGCCTTGCCCGTTGGCTTCGTCCTGGTGCGCTCCTTCTTCTTCTTGGTCGTCGGCTTCTTCTTGTCCCGCGCCGTGGTGTTGTTCGTGGGCGTCTCCCTGGTGTTCCGCTTCGTGCTGTTGGGCTTCGTCTTCTTGGTGGTCGGCTTCTTCCTGGGGCTCTTGCTGGTGTTCTTCTTCTTCTGCCTCTTCGTCTTTTTCTTGGCCGTCTTGCTGTTGTTCGGCTTCTTGGTGTTCTTCTT-3′; boldface letters denote the sequence of T7 class II promotor (ϕ2.5)) and (anti-sense: 5′-AAGAAGAACACCAAGAAGCCGAACAACAGCAAGACGGCCAAGAAAAAGACGAAGAGGCAGAAGAAGAAGAACACCAGCAAGAGCCCCAGGAAGAAGCCGACCACCAAGAAGACGAAGCCCAACAGCACGAAGCGGAACACCAGGGAGACGCCCACGAACAACACCACGGCGCGGGACAAGAAGAAGCCGACGACCAAGAAGAAGAAGGAGCGCACCAGGACGAAGCCAACGGGCAAGGCGGACAAGAAGAAGACGAGCAGCAACAAGAGGACGGGGAAGCGGCAGAAGCACAGCACGCCGAAGGACAGGGAGGACACGAGGGAGGGCCAGGGCACGGGCAGCAAGCCGGAGGAGCAGAAGAACAACAGGGACAGCAAGCCGAAGGAGGCAACGCCCACGCCCACGCCGGACACGCAGAACAAGAGGCCGAAAACGACGCCGACCAGCACGACCAGGAAGGGCACCACCCCGGAGAACAGCACCACGCCCAAGCACACCATAATAGTGAGTCGTATTA-3′). For *in vitro* transcription, 10 μM of double-stranded DNA (dsDNA) template in 100 μl transcription buffer (Promega, catalog: P1300), along with 1 mM of each of GTP, CTP and UTP, with 1mM ATP (for normal RNA), 4 mM NAD (for NAD-RNA) or 4 mM m^7^GpppA (New England Biolabs, catalog: S1406S) (for m^7^G-RNA), 10 μl of T7 RNA polymerase (Promega, catalog: P1300), 5% DMSO, 5 mM DTT and 2.5-unit RNase inhibitor were added and the transcription mixture was incubated at 37°C for 4 h. The reaction was incubated with 11-unit DNase I (Promega, catalog: P1300) at 37°C for 30 min to remove the DNA template. RNA was then extracted using acid phenol/chloroform and precipitated with isopropanol (with 0.3 M sodium acetate, pH 5.5) at -80°C overnight. The RNA pellet was washed twice with 75% ethanol, air-dried, redissolved in DEPC-treated H_2_O, and stored at –80°C. For spike-in RNAs used as internal controls for gene-specific qRT-PCR assessment, NAD-RNA is 106 nt as described above. To prepare ppp-RNA, 88 nt oligo nucleotide was synthesized (Genewiz, China) and annealed to make double-stranded DNA template. Primer extension was used to increase the length of the template to encode 106 nt RNA (template sequence: 5′-GATCAC**TAATACGACTCACTATT**GGATACCGGGAAAACGCTGGGCGTTAATCAAAGAGGCGAACTGTGTGTGAGAGGTCCTATGATTATGTCCGGTTATGTAAACAATCCGGAAGCGACCAACGCCTTG-3′; boldface letters denote the sequence of T7 class II promotor (ϕ2.5)) and (anti-sense: 5′-CAAGGCGTTGGTCGCTTCCGGATTGTTTACATAACCGGACATAATCATAGGACCTCTCACACACAGTTCGCCTCTTTGATTAACGCCCAGCGTTTTCCCGGTATCCAATAGTGAGTCGTATTAGTGATC-3′). To prepare 45 nt m^7^Gppp-RNA, oligo nucleotide was synthesized (Genewiz) and annealed to make double-stranded DNA template. Primer extension was used to increase the length of the template to encode 45 nt RNA (template sequence: 5′-GATCAC**TAATACGACTCACTATT**ACTGTGTCGTCGTCGTCTGCTGTCTCTCTCTCGCGGGCCCTGTCG-3′; boldface letters denote the sequence of T7 class II promotor (ϕ2.5)) and (anti-sense: 5′-CGACAGGGCCCGCGAGAGAGAGACAGCAGACGACGACGACACAGTAATAGTGAGTCGTATTAGTGATC-3′).

For boronate affinity experiment, we synthesized m^7^G-RNA (500 nt) and ppp-RNA (500 nt) spike-ins with different sequences from that of NAD-RNA (500 nt, see above). The synthesis of m^7^G-RNA (500 nt) was as described in (17). To prepare m^7^G-RNA, oligonucleotide was synthesized (Genewiz) and were subjected to polyadenylation for poly(A) tails elongation (template sequence: 5′-**GATCACTAATACGACTCACTATTAC**ATGGAGGGCTCCGTGAACGGCCACGAGTTCGAGATCGAGGGCGAGGGCGAGGGCCGCCCCTACGAGGGCACCCAGACCGCCAAGCTGAAGGTGACCAAGGGTGGCCCCCTGCCCTTCGCCTGGGACATCCTGTCCCCTCAGTTCATGTACGGCTCCAAGGCCTACGTGAAGCACCCCGCCGACATCCCCGACTACTTGAAGCTGTCCTTCCCCGAGGGCTTCAAGTGGGAGCGCGTGATGAACTTCGAGGACGGCGGCGTGGTGACCGTGACCCAGGACTCCTCCCTGCAGGACGGCGAGTTCATCTACAAGGTGAAGCTGCGCGGCACCAACTTCCCCTCCGACGGCCCCGTAATGCAGAAGAAGACCATGGGCTGGGAGGCCTCCTCCGAGCGGATGTACCCCGAGGACGGCGCCCTGAAGGGCGAGATCAAGCAGAGGCTGAAGCTGAAGGACGGCGGCCACTACGACGCTGAGGTCAAGACCACCTACAAGGCCA-3′; boldface letters denote the sequence of T7 class II promotor (ϕ2.5)) and (anti-sense: 5′- TGGCCTTGTAGGTGGTCTTGACCTCAGCGTCGTAGTGGCCGCCGTCCTTCAGCTTCAGCCTCTGCTTGATCTCGCCCTTCAGGGCGCCGTCCTCGGGGTACATCCGCTCGGAGGAGGCCTCCCAGCCCATGGTCTTCTTCTGCATTACGGGGCCGTCGGAGGGGAAGTTGGTGCCGCGCAGCTTCACCTTGTAGATGAACTCGCCGTCCTGCAGGGAGGAGTCCTGGGTCACGGTCACCACGCCGCCGTCCTCGAAGTTCATCACGCGCTCCCACTTGAAGCCCTCGGGGAAGGACAGCTTCAAGTAGTCGGGGATGTCGGCGGGGTGCTTCACGTAGGCCTTGGAGCCGTACATGAACTGAGGGGACAGGATGTCCCAGGCGAAGGGCAGGGGGCCACCCTTGGTCACCTTCAGCTTGGCGGTCTGGGTGCCCTCGTAGGGGCGGCCCTCGCCCTCGCCCTCGATCTCGAACTCGTGGCCGTTCACGGAGCCCTCCATGTAATAGTGAGTCGTATTAGTGATC-3′). To prepare ppp-RNA, oligonucleotide was synthesized (Genewiz) and were subjected to polyadenylation for poly(A) tails elongation (template sequence: 5′-**TAATACGACTCACTATT**ATCTGCACCACCGGCAAGCTGCCCGTGCCCTGGCCCACCCTCGTGACCACCCTGACCTACGGCGTGCAGTGCTTCAGCCGCTACCCCGACCACATGAAGCAGCACGACTTCTTCAAGTCCGCCATGCCCGAAGGCTACGTCCAGGAGCGCACCATCTTCTTCAAGGACGACGGCAACTACAAGACCCGCGCCGAGGTGAAGTTCGAGGGCGACACCCTGGTGAACCGCATCGAGCTGAAGGGCATCGACTTCAAGGAGGACGGCAACATCCTGGGGCACAAGCTGGAGTACAACTACAACAGCCACAACGTCTATATCATGGCCGACAAGCAGAAGAACGGCATCAAGGTGAACTTCAAGATCCGCCACAACATCGAGGACGGCAGCGTGCAGCTCGCCGACCACTACCAGCAGAACACCCCCATCGGCGACGGCCCCGTGCTGCTGCCCGACAACCACTACCTGAGCACCCAGTCCGCCCTGAGCAAAGACCCCAACGA-3′; boldface letters denote the sequence of T7 class II promotor (ϕ2.5)) and (anti-sense: 5′-TCGTTGGGGTCTTTGCTCAGGGCGGACTGGGTGCTCAGGTAGTGGTTGTCGGGCAGCAGCACGGGGCCGTCGCCGATGGGGGTGTTCTGCTGGTAGTGGTCGGCGAGCTGCACGCTGCCGTCCTCGATGTTGTGGCGGATCTTGAAGTTCACCTTGATGCCGTTCTTCTGCTTGTCGGCCATGATATAGACGTTGTGGCTGTTGTAGTTGTACTCCAGCTTGTGCCCCAGGATGTTGCCGTCCTCCTTGAAGTCGATGCCCTTCAGCTCGATGCGGTTCACCAGGGTGTCGCCCTCGAACTTCACCTCGGCGCGGGTCTTGTAGTTGCCGTCGTCCTTGAAGAAGATGGTGCGCTCCTGGACGTAGCCTTCGGGCATGGCGGACTTGAAGAAGTCGTGCTGCTTCATGTGGTCGGGGTAGCGGCTGAAGCACTGCACGCCGTAGGTCAGGGTGGTCACGAGGGTGGGCCAGGGCACGGGCAGCTTGCCGGTGGTGCAGATAATAGTGAGTCGTATTA-3′).

### HEEB reaction with NAD-RNA (38 nt)

NAD-RNA (1 μg) was reacted with 40 mM and 100 mM HEEB (1 M stock in DMSO) under the catalysis of ADPRC (25 μg/ml) with 0.4 U/μl of RNase Inhibitor (Takara Bio, catalog: 2313A) in 100 μl of ADPRC reaction buffer (50 mM Na-HEPES pH 7.0, 5 mM MgCl_2_) at 37°C for 1 h. Product was purified with Zymo column (RNA Clean & Concentrator-5, Zymo Research, catalog: R1016) according to the instruction of manufacturer and analyzed by an 8% polyacrylamide TBE urea gel. Gel was stained by SYBR Gold (Invitrogen, catalog: S11494) and fluorescence was detected by Typhoon FLA 7000 fluorescent image analyzer (GE Life Science).

### Enrichment of biotinylated NAD-RNA (38 nt) and m^7^Gppp-RNA (38 nt)

Magnetic streptavidin beads (6 μl, MedChemExpress, catalog: HY-K0208) were washed 3 times with immobilization buffer (10 mM Na-HEPES, 1 M NaCl, 5 mM EDTA) (17), and then incubated with the purified RNA product from HEEB reaction in immobilization buffer, together with 0.4 U/μl of RNase Inhibitor, in a thermomixer at room temperature for 30 min. After the beads were washed 5 times with streptavidin wash buffer (50 mM Tris–HCl, pH 7.4, 8 M urea) (17), the biotinylated RNA on the beads was extracted with Trizol LS (Ambion, catalog: 10296010) and chloroform. The upper aqueous layer was further purified with Zymo column (Zymo Research, catalog: R1016). The purified RNA product was analyzed by an 8% polyacrylamide TBE urea PAGE gel. Gel was stained by SYBR Gold and fluorescence was detected by Typhoon FLA 7000 fluorescent image analyzer (GE Life Science).

### Dot blot analysis

500 ng RNA was blotted on Hybond N + membrane (GE Healthcare, catalog: RPN203B) which was then cross-linked by UV_254nm_ (0.18 J/cm^2^) twice. Membrane was blocked in 5% BSA in PBST (0.1% Tween20 in PBS) for 1 h, followed by incubation with Alexa Fluor® 790 Streptavidin (Jackson, catalog: 016–650-084, 1:10 000 in PBST) at room temperature for 2 h in the dark. Membrane was washed with PBST and PBS for 3 times, respectively, and imaged on the Odyssey LiCor CLx scanner (Li-Cor Biosciences) with the software set to auto-detect the signal intensity at 800 nm channel. Finally, the membrane was stained with methylene blue solution (0.3% w/v methylene blue + 30% v/v ethanol + 70% v/v H_2_O) (19) to visualize the presence of RNA.

### HEEB reaction for NAD-RNA (38 nt) and m^7^Gppp-RNA (45 nt)

100 ng of spike-in NAD-RNA (38 nt) and 200 ng of m^7^Gppp-RNA (45 nt) were incubated with 100 mM HEEB (1 M stock in DMSO) with ADPRC (25 μg/ml) in 100 μl of ADPRC reaction buffer (50 mM Na-HEPES pH 7.0, 5 mM MgCl_2_) at 37°C for 1 h. 100 μl of DEPC-treated H_2_O was then added and acid phenol/ether extraction was performed to stop the reaction (17). RNAs were precipitated by ethanol, re-dissolved in 20 μl of DEPC-treated H_2_O. 5 μl of biotinylated RNAs were kept as input. After HEEB reaction, biotinylated RNAs were incubated with streptavidin bead particles (6 μl, MedChemExpress, catalog: HY-K0208) and 0.4 U/μl of RNase Inhibitor (Takara Bio, catalog: 2313B) at 25°C for 30 min. Beads were washed four times with streptavidin wash buffer (50 mM Tris–HCl (pH 7.4) and 8 M urea), and three times with DEPC-treated H_2_O. The biotinylated RNA on the beads was extracted with Trizol LS (Ambion, catalog: 10296010) and chloroform. Input (see above) RNAs and the biotinylated RNA on the beads were analyzed by an 8% polyacrylamide TBE urea PAGE gel. Gel was stained by SYBR Gold and fluorescence was detected by Typhoon FLA 7000 fluorescent image analyzer (GE Life Science).

### NudC treatment of NAD-RNA (38 nt) and m^7^Gppp-RNA (38 nt)

De-capping of 200 ng NAD-RNA (38 nt) or m^7^Gppp-RNA (38 nt) was performed with 1 μl of NudC (New England Biolabs, catalog: M0607S) in 25 μl of NudC reaction buffer (100 mM NaCl, 50 mM Tris–HCl pH 7.9, 10 mM MgCl_2_, 100 μg/ml Recombinant Albumin) at 37°C for 30 min. Product was purified with Trizol LS (Ambion, catalog: 10296010) and analyzed by an 8% polyacrylamide TBE–urea gel. Gel was stained by SYBR Gold (Invitrogen, catalog: S11494) and fluorescence was detected by Typhoon FLA 7000 fluorescent image analyzer (GE Life Science).

### NudC treatment of NAD-RNA (38 nt) and m^7^Gppp-RNA (45 nt)

100 ng of spike-in NAD-RNA (38 nt) and 200 ng of m^7^Gppp-RNA (45 nt) were mixed together and incubated with 500 nM NudC (New England Biolabs, catalog: M0607S) in 25 μl of NudC reaction buffer (100 mM NaCl, 50 mM Tris–HCl pH 7.9, 10 mM MgCl_2_, 100 μg/ml Recombinant Albumin) at 37°C for 30 min. Product was purified with Trizol LS (Ambion, catalog: 10296010) and analyzed by an 8% polyacrylamide TBE urea gel. Gel was stained by SYBR Gold (Invitrogen, catalog: S11494) and fluorescence was detected by Typhoon FLA 7000 fluorescent image analyzer (GE Life Science).

### HEEB reaction with total RNA extract

Total RNA was prepared from mouse liver tissues, in accordance with manufacturer's instruction (Takara Bio, catalog: 9108). Total RNAs (100 μg) was incubated with 100 mM HEEB (1 M stock in DMSO), ADPRC (25 μg/ml, Sigma-Aldrich, catalog: A9106) and 0.4 U/μl of RNase Inhibitor (Takara Bio, catalog: 2313B) in 100 μl of ADPRC reaction buffer at 37°C for 1 h. 100 μl of DEPC-treated H_2_O was then added and acid phenol/ether extraction was performed to stop the reaction (17). RNAs were precipitated by ethanol, re-dissolved in 100 μl of DEPC-treated H_2_O. 5 μl of biotinylated RNAs were kept as input.

### NudC-catalyzed NAD-RNA elution

After HEEB reaction, biotinylated RNAs were incubated with streptavidin bead particles (6 μl, MedChemExpress, catalog: HY-K0208) and 0.4 U/μl of RNase Inhibitor (Takara Bio, catalog: 2313B) at 25°C for 30 min. Beads were washed four times with streptavidin wash buffer (50 mM Tris–HCl (pH 7.4) and 8 M urea), and three times with DEPC-treated H_2_O. To ensure complete elution, biotin-conjugated RNAs were replaced from streptavidin beads by incubating with 1 mM biotin buffer (20 μl, Sigma-Aldrich, catalog: B4639) at 94°C for 8 min, followed by incubation with 500 nM NudC (New England Biolabs, catalog: M0607S) in 25 μl of NudC reaction buffer (100 mM NaCl, 50 mM Tris–HCl pH 7.9, 10 mM MgCl_2_, 100 μg/ml Recombinant Albumin) at 37°C for 30 min. After NudC treatment, biotinylated-RNAs that are resistant to NudC catalysis, potentially derived from contaminating m^7^G-RNAs, were retained on beads by incubation with high-capacity streptavidin particle (20 μl, Thermo Fisher Scientific, catalog: 20357) at 25°C for 30 min. Eluted RNAs in the supernatant were used for next step.

### yDcpS treatment of NAD-RNA (38 nt) and m^7^Gppp-RNA (38 nt)

De-capping of 200 ng NAD-RNA (38 nt) and m^7^Gppp-RNA (38 nt) was performed with or without 1 μl of yDcpS (New England Biolabs, catalog: M0463S) in 1X yDcpS reaction buffer (10 mM Bis-Tris–HCl pH 6.5, 1 mM EDTA) in 20 μl total volume at 37°C for 1 h. Product was purified with Zymo column (RNA Clean & Concentrator-5, Zymo Research, catalog: R1016) according to the instruction of manufacturer and analyzed by an 8% polyacrylamide TBE urea gel. Gel was stained by SYBR Gold (Invitrogen, catalog: S11494) and fluorescence was detected by Typhoon FLA 7000 fluorescent image analyzer (GE Life Science).

### qRT-PCR for ppp-RNA (106 nt), NAD-RNA (106 nt) and m^7^Gppp-RNA (106 nt)

Total RNAs (100 μg) and 100 ng of spike-in NAD-RNA (106 nt), ppp-RNA (106 nt) or m^7^Gppp-RNA (106 nt) were incubated with 100 mM HEEB (1 M stock in DMSO) with or without ADPRC (25 μg/ml) in 100 μl of ADPRC reaction buffer (50 mM Na-HEPES pH 7.0, 5mM MgCl_2_) at 37°C for 1 h, followed by NudC-catalyzed NAD-RNA elution. Input (see above) and NudC-eluted RNAs from three biological replicates were used for qRT-PCR. Reverse transcription was performed with gene specific primers using SuperScript III SuperMix (Vazyme, catalog: R323-01). qPCR was performed using SYBR Green master mix (Vazyme, catalog: Q111-02) to detect NAD-RNA, ppp-RNA or m^7^Gppp-RNA from three biological replicates using specific primers described in Supplementary Table S4. Significance was assessed by Student's *t* test.

### PolyA-selected RNA sequencing

Input (see above) and NudC-eluted RNAs from four biological replicates of livers from 2- and 18-month old C57BL/6 mice were used for NGS library construction, in accordance with manufacturer's instructions (mRNA-seq Lib Prep Kit for Illumina, Abclonal, catalog: RK20302). Library quality was assessed by Bioanalyzer 2100 (Agilent, United States), and quantification was performed by qRT-PCR with a reference to a standard library. Libraries were pooled together in equimolar amounts to a final 2 nM concentration and denatured with 0.1 M NaOH (Sigma, catalog: 72068). Libraries were sequenced on the Illumina NovaSeq 6000 system (paired end; 150 bp).

### ONE-seq with polyadenylated NAD-RNA (500 nt) and m^7^Gppp-RNA (500 nt)

Total RNAs (100 μg) were mixed with 1 ng of 3′-end polyadenylated spike-in RNA (500 nt) that had 0% NAD-RNA/100% m^7^Gppp-RNA, 1% NAD-RNA/99% m^7^Gppp-RNA, 5% NAD-RNA/95% m^7^Gppp-RNA or 10% NAD-RNA/90% m^7^Gppp-RNA, respectively. The mixture of total RNAs and spike-in RNA (500 nt) were incubated with 100 mM HEEB (1 M stock in DMSO) with ADPRC (25 μg/ml) in 100 μl of ADPRC reaction buffer (50 mM Na-HEPES pH 7.0, 5 mM MgCl_2_) at 37°C for 1 h, followed by NudC-catalyzed NAD-RNA elution. Input (see above) and NudC-eluted RNAs from three biological replicates of livers from 18-month old C57BL/6 mice were subjected to polyA-selected RNA sequencing as mentioned above.

### 3′-End blocking by adaptor ligation

1 μg of NAD-RNA (38 nt) or m^7^Gppp-RNA (38 nt) was ligated with 5 μM 3′ adaptor oligo listed in Supplementary Table S5, in the presence of 10 U T4 RNA ligase 1 (New England Biolabs, catalog: M0202), 10% of PEG8000, 1 mM ATP and 40U RNaseOUT in 20 μl of 1x T4 RNA ligase buffer. Reaction was incubated at 16°C for 16 h. RNAs were purified by Trizol LS (Ambion, catalog: 10296010) according to the instruction of manufacturer and analyzed by an 8% polyacrylamide TBE urea gel. Gel was stained by SYBR Gold (Invitrogen, catalog: S11494) and fluorescence was detected by Typhoon FLA 7000 fluorescent image analyzer (GE Life Science).

### Enrichment of RNA oligonucleotide by boronic acid beads

Boronic acid beads (30 μl, Smart-Lifesciences, catalog: SA057005) were washed 3 times with binding buffer (50 mM Na-HEPES, 1 M NaCl, PH 7.8), and then incubated with RNA oligonucleotide in binding buffer in a thermomixer at 37°C for 2 h. Beads were then washed 3 times with wash buffer (50 mM PBS, pH 7.4, 6 M urea); RNA oligonucleotide bound by the beads was extracted with Trizol LS (Ambion, catalog: 10296010)). Purified RNA product was analyzed by an 8% polyacrylamide TBE–urea PAGE gel. Gel was stained by SYBR Gold and fluorescence was detected by Typhoon FLA 7000 fluorescent image analyzer (GE Life Science).

### qRT-PCR analysis of NAD-capped RNAs bound by boronic acid beads

Total RNAs were extracted from three biological replicates of livers from 2-month old C57BL/6 mice as described above. For each replicate, total RNAs (50 μg) were mixed with polyadenylated spike-in RNAs (1 ng NAD-RNA, 1 ng m^7^Gppp-RNA, and 1 ng of ppp-RNA). RNAs were incubated with yDcpS (New England Biolabs, catalog: M0463) in 50 μl of 1X yDcpS reaction buffer at 37°C for 1 h, to remove m^7^G cap. After purification with Trizol LS (Ambion, catalog: 10296010), yDcpS-treated RNAs were combined with 100 μmol oligo d(T)30VN in 100 μl 0.5× SSC buffer (Sangon Biotech, catalog: M0463), and incubated at 80°C for 5 min followed by 50°C for 60 min. Then, 1 μl RNase H (Thermo Fisher Scientific, catalog: EN0202) and 5.6 μl 10× Reaction buffer (Thermo Fisher Scientific, catalog: EN0202) were added. The mixture was incubated at 37°C for 20 min, and purified with Trizol LS (Ambion, catalog: 10296010). This step aims to promote 3′-end adaptor ligation by removing polyA sequence tract from the endogenous transcripts. Prior to 3′-end ligation, polyA-depleted RNAs were treated with 2 U CIAP (Thermo Fisher Scientific, catalog: 18009019) in the presence of 40 U RNaseOUT at 37°C for 1 h to remove 5′-terminal phosphate. RNA was extracted with Trizol LS (Ambion, catalog: 10296010). 5 μg of CIAP-treated RNA products were ligated with 100 μM 3′ adaptor oligo listed in Supplementary Table S5, in the presence of 10 U T4 RNA ligase 1 (New England Biolabs, catalog: M0202), 10% of PEG8000, 1 mM ATP and 40 U RNaseOUT in 50 μl of 1× T4 RNA ligase buffer. Reaction was incubated at 16°C for 16 h. RNAs were purified by Trizol LS (Ambion, catalog: 10296010), and re-dissolved in 100 μl of binding buffer (50 mM Na-HEPES, 1 M NaCl, PH 7.8). RNAs were incubated with boronic acid beads (30 μl, Smart-Lifesciences, catalog: SA057005) at 37°C for 2 h. Beads were washed three times with wash buffer (50 mM PBS and 6 M urea), and once with DEPC-treated H_2_O. Reverse transcription was performed with adaptor-based primer listed in Supplementary Table S5, by BeyoRT™ II First-Strand cDNA Synthesis Kit (Beyotime, catalog: D7168M). Meanwhile, 5 μg of CIAP-treated RNAs without 3′ ligation were incubated with boronic acid beads, reverse transcribed with random hexamer primers, and served as input. After reverse transcription, DNA/RNA mixtures were treated with 1 U RNase H at 37°C for 20 min. While ppp-RNA (500 nt) was served as the baseline, NAD-RNA (500 nt) was used as a positive control, and m^7^G-RNA (500 nt) was used as a negative control. Primers were listed in Supplementary Table S4. To calculate the fold enrichment of each adaptor + reaction from qPCR data, we normalized the Ct value of the target RNA to the Ct of the ppp-RNA. Fold enrichments were calculated by normalizing adaptor + fraction value (ΔCt of target RNA normalized to the ppp-RNA) to nonspecific background (the similar ΔCt calculation of the input), to yield the ΔΔCt value. The linear conversion of this ΔΔCt resulted in fold enrichment. Significance was assessed by Student's *t-*test.

### Data analysis

All sequencing reads were processed with Trim Galore (v0.6.6) (20) with the parameters ‘–nextseq 30 –paired’ to remove the adapter sequences (AGATCGGAAGAGC) from NovaSeq-platforms, and reads longer than 20 bp were kept. Reads that passed the quality control procedure were kept and mapped to the Mus musculus genome (GRCm38) using STAR (2.7.6a) (21) with default parameters. Mapped read pairs were counted against Gencode (M23) annotations using featureCounts (v2.0.1) with parameters ‘-p -B -C’ (22). Aligned reads were summarized as exon counts and gene body counts using exon annotation and gene body annotation, respectively. Intron read counts were calculated by subtracting exon counts from gene body counts at gene-level (23). Read alignments were converted to the bigwig files by bamCoverage (24) and were visualized at selected genomic regions by IGV (v2.8.3) (25). Sequencing saturation was assessed by randomly subsampling of the original libraries and analysis of the corresponding changes in gene numbers with more than 10 read counts. The gene body coverage of sequencing reads was calculated using RSeQC (26) by counting the number of reads covering each nucleotide position and all transcripts were scaled to 100 bins.

Genes that had zero count in more than 25% (3 out of 12) sequencing libraries were removed before performing differential analysis. Principle component analysis (PCA) was performed with R function ‘prcomp’ using a transformed counts matrix by function ‘vst’. To identify NAD-RNA from total RNA-seq data, we used R package *DESeq2* (v1.30.0) (27) to perform differential analysis. Significance of logarithmic fold changes were determined by a Wald test to approximate *P* values, and genes passing an independent filtering step were adjusted for multiple testing using the Benjamini-Hochberg procedure to yield a false discovery rate (FDR). NAD-RNAs were defined as fold change of normalized transcript counts ≥2 and FDR <0.05 in NudC-treated samples compared to those in input samples. Gene annotation information, such as chromosome, gene-types, gene-lengths and intron coordinates were retrieved from Gencode (M23) annotations. The violin plot, bar plot, line chart and scatter plot were generated by R package *ggplot2* (v3.3.2) (28).

Pathway enrichment analysis was performed using R package *gprofiler2* (v0.2.1) (29). Pathways were defined as the molecular pathways of Reactome (BioMart releases: 2021-5-7) (30) and the biological processes (BPs) of GO (BioMart releases: 2021-05-01) (31) without less reliable GO annotations (IEAs) that are not manually reviewed. Size of enriched gene sets were limited to range between 5 and 350. Multiple testing corrections were performed with *gprofiler2* built-in ‘g_SCS’ method and terms with adjusted *P*-value <0.05 were considered as significantly enriched. Top 10 significantly enriched pathways were visualized in bar plot. In addition, the results of the pathway analysis were visualized using the EnrichmentMap App (v1.1.0) in Cytoscape (v3.8.2) (32). Network maps were generated for nodes with FDR <0.05 and nodes sharing gene overlaps with Jaccard coefficient >0.60 were connected by a blue line (edge). Clusters of related pathways were identified and annotated using the AutoAnnotate App (v1.3.5) in Cytoscape that uses a Markov Cluster (MCL) algorithm which connects pathways by shared keyword in the description of each pathway. The resulting clusters of pathways were manually reviewed and were designated as the major pathways in a circle (33).

### ONE-seq-based gene specific analysis of NAD capping

Total RNA was extracted from four biological replicates of livers from 2-month old C57BL/6 mice as described above. For each replicate, 100 μg total RNA, mixed with 1 ng of NAD-RNA (106 nt) and 1 ng of ppp-RNA (106 nt), was incubated with 100 mM HEEB (1 M stock in DMSO) with ADPRC (25 μg/ml) in 100 μl of ADPRC reaction buffer (50 mM Na-HEPES pH 7.0, 5 mM MgCl_2_) at 37°C for 1 h. 100 μl of DEPC-treated H_2_O was then added and acid phenol/ether extraction was performed to stop the reaction (17). RNAs were precipitated by ethanol, re-dissolved in 100 μl of DEPC-treated H_2_O. 1 μl of biotinylated RNAs were kept as input. After NudC-catalyzed NAD-RNA elution, input and NudC-eluted RNAs were used for reverse transcription. Quantification of transcript abundance by qRT-PCR was performed using SYBR Green master mix (Vazyme, catalog: Q111-02) following the manufacturer's protocol with primers listed in Supplementary Table S4. ppp-RNA (106 nt) was served as the baseline, and NAD-RNA (106 nt) was used as an internal positive control. To calculate the enrichment from qRT-PCR data, the Ct value of the target gene was first normalized to the Ct of the ppp-RNA (baseline). Next, the normalized NudC + fraction value (ΔCt of the target gene normalized to the ppp-RNA) was normalized to the background (ΔCt calculation for the gene in the input), to yield the ΔΔCt value. The linear conversion of this ΔΔCt resulted in the fold enrichment.

## RESULTS

### The workflow of ONE-seq

Compared to previous methods that require multiple reactions, we introduced HEEB (*N*-[2-(2-hydroxyethoxy) ethyl]-biotinamide) as a reactant, allowing only one reaction to biotinylate NAD-capped RNAs. To avoid contaminating signals, we designed a NudC-based post-treatment to elute biotin-conjugated RNAs specifically derived from NAD, but not m^7^G-capped transcripts from streptavidin beads. Therefore, our method circumvented laborious steps to purify mRNAs followed by clearance of m^7^G-capped RNAs. With significantly simplified procedures, our method enabled NAD-RNA profiling directly from total RNA. Based on the same platform, qRT-PCR analysis on specific NAD-RNAs could be readily applied.

**Figure 1.**
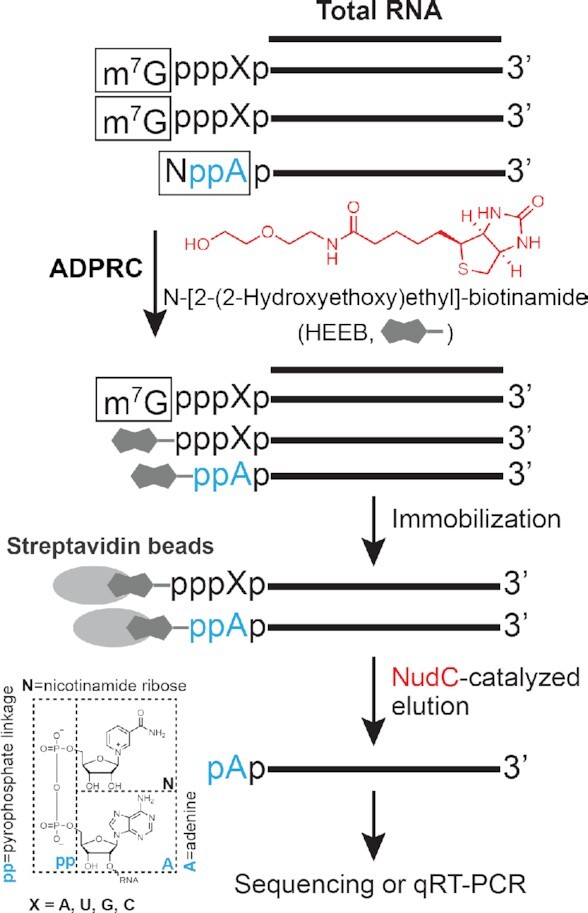
The workflow of ONE-seq. HEEB (*N*-[2-(2-hydroxyethoxy) ethyl]-biotinamide, in red) has a terminal hydroxyl group as the nucleophile and a biotin group as the affinity tag. In the presence of ADPRC, nicotinamide moiety of NAD can be replaced by HEEB via nucleophilic reaction and simultaneously biotinylated, which subsequently can be enriched by streptavidin beads. NudC (in red) detaches HEEB-RNAs specifically derived from NAD-capped transcripts from streptavidin beads, while contaminating m^7^G-RNAs that also react with HEEB remain retained on the bead. Eluted RNAs can be used for epitranscriptome-wide profiling as well as gene-specific qRT-PCR analysis.

We thereby named our method ONE-seq, through **O**ne-step chemo-enzymatic reaction to biotinylate NAD-capped RNA for streptavidin binding, followed by the nudix phosphohydrolase **N**udC-catalyzed **E**lution, to specifically harvest NAD-capped RNA from streptavidin beads for high-throughput **seq**uencing (Figure 1). First, HEEB has a terminal hydroxyl group as the nucleophile and a biotin group as the affinity tag. Catalyzed by ADPRC, nicotinamide moiety of NAD can be replaced by HEEB via nucleophilic reaction and simultaneously biotinylated, which subsequently can be enriched by streptavidin beads. Second, NudC, known for its ability to hydrolyze the diphosphate, but not triphosphate, linkage, detaches HEEB-RNAs specifically derived from NAD-capped transcripts from streptavidin beads. At this step, contaminating m^7^G-RNAs that also react with HEEB are retained on the bead. Third, eluted RNAs can be used for epitranscriptome-wide profiling as well as gene-specific qRT-PCR analysis.

### The feasibility of one-step chemo-enzymatic reaction

**Figure 2.**
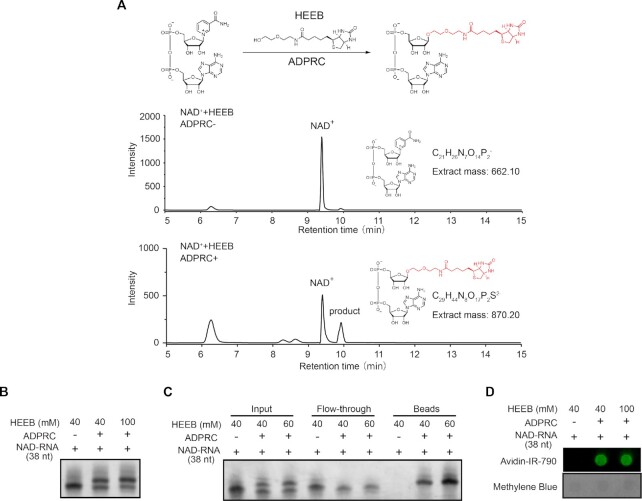
The feasibility of one-step chemo-enzymatic reaction. (**A**) HPLC spectra confirm a product corresponding to the biotinylated NAD-derived structure. Top panel: chemical reaction between NAD molecule and HEEB; middle panel: HPLC spectrum of NAD, and bottom panel: HPLC spectrum of HEEB-reacted NAD. (**B**) HEEB reacts with NAD-RNAs (38 nt) in an ADPRC-dependent manner, as evidenced by the accumulation of an upper band in the TBE-UREA gel. (**C**) Biotinylated NAD-RNAs (38 nt) are retained on streptavidin beads, with the lower band of non-biotinylated form being discarded with flow-through. (**D**) Biotinylation is detected by Avidin-IR-790, an avidin-conjugated fluorophore. Methylene blue indicated loading control for the dot blot.

**Figure 3.**
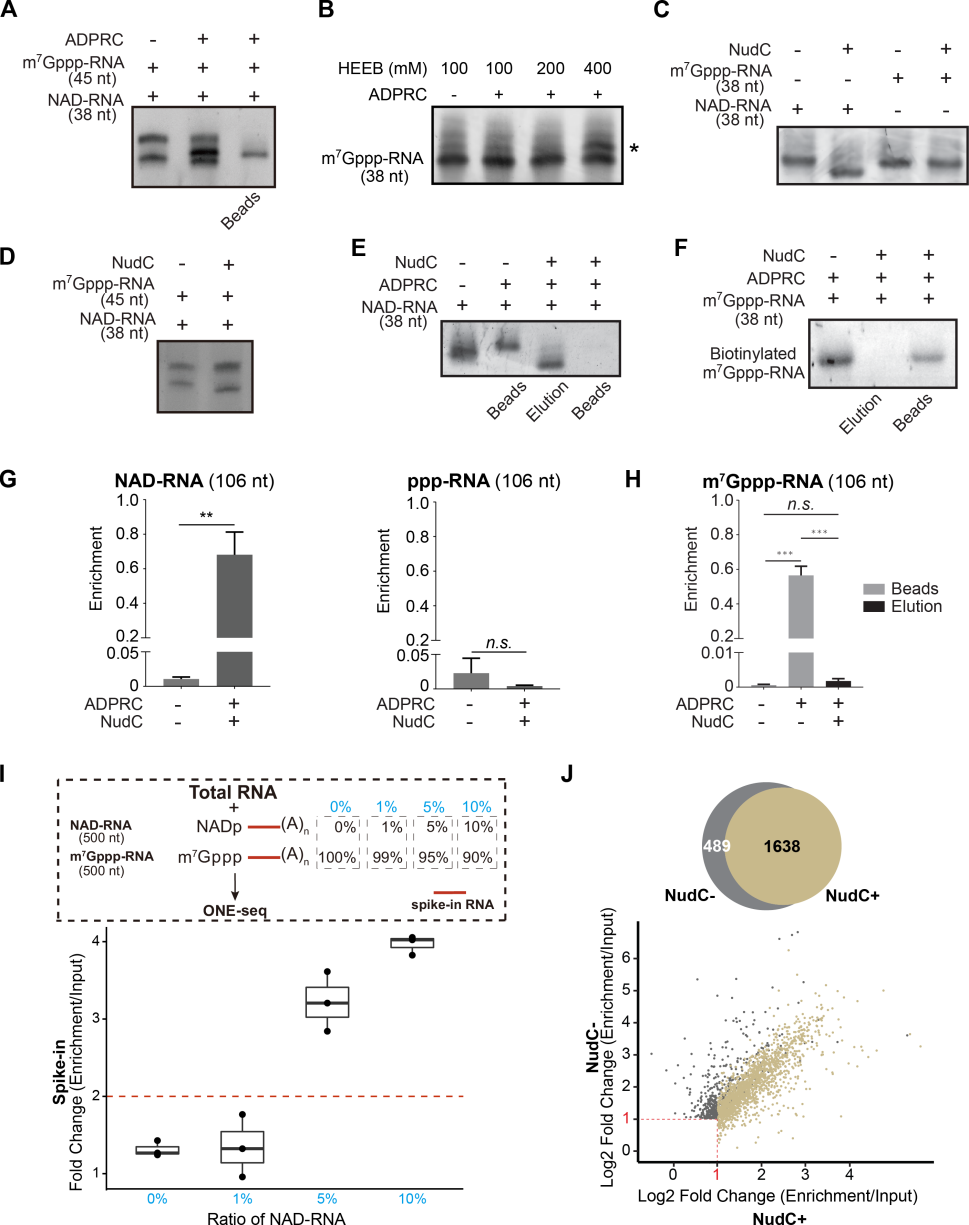
The validation of ONE-seq. (**A**) HEEB reacts with NAD-RNA (38 nt), but not m^7^Gppp-RNA (45 nt), to yield a biotinylated form. (**B**) HEEB, only at high concentration (400 mM), reacts with m^7^G-capped RNA (38 nt), as evidenced by an upper band in the TBE-UREA gel (marked by asterisk). (**C**) NudC can de-cap NAD-RNA (38 nt), but not m^7^G-capped RNA (38 nt), as shown by a lower-sized band corresponding to the de-capped product in the TBE-UREA gel. (**D**) In the same reaction, NudC was able to selectively de-cap NAD-capped (38 nt) but not m^7^G-capped RNA (45 nt). (**E**) NudC-mediated de-capping elutes NAD-RNA (38 nt) from streptavidin beads. (**F**) NudC cannot elute biotinylated m^7^G-RNA (38 nt) from the streptavidin beads. (**G**) qRT-PCR analysis shows that NAD-RNA (106 nt), but not ppp-RNA (106 nt), can be enriched by ONE-seq. (**H**) qRT-PCR analysis shows that streptavidin beads bound HEEB-reacted m^7^G-capped RNA (106 nt) cannot be eluted by NudC (Two-tailed Student's *t* test: ***P < 0.001; **P < 0.01; *n.s*., not significant). (**I**) RNA-seq experiment of spike-in RNAs determines the sensitivity of ONE-seq. Top panel: schematic workflow of total RNAs and polyadenylated spike-in RNAs that had different ratios of NAD-RNA (500 nt). Two spike-in RNAs with identical sequence (500 nt) but have either NAD or m^7^G-cap, followed by polyA tails are used; bottom panel: fold change of normalized read counts from spike-in RNA between enrichment and input samples in different ratios of NAD-RNA. Total RNAs were from liver tissues of 18-month mice. The nominal ratios of NAD-RNA were highlighted in blue. (**J**) Epitranscriptome assessment of NudC to minimize the noise of m^7^G-RNAs. Two-fold enrichment of read counts was used as the cutoff. Standard ONE-seq identified 1,638 NAD-RNAs, while 489 false-positive NAD-RNAs were found without the use of NudC, presumably derived from m^7^G-capped RNAs. Total RNAs were from liver tissues of 12-month mice.

We tested the feasibility of one-step chemo-enzymatic reaction. First, we performed an ADPRC catalyzed reaction between NAD molecule and HEEB. HPLC and LC–MS confirmed a product corresponding to the biotinylated NAD-derived structure (Figure 2A and Supplementary Figure S1). Second, we subjected a synthetic 38-nucleotide (nt) NAD-capped RNA to the HEEB reaction, resulting in an ADPRC-dependent yielding, evidenced by the accumulation of an upper band in the TBE-UREA gel (Figure 2B). By incubation with the streptavidin beads, we showed the evidence that only the upper band from the reaction was retained by the streptavidin beads, while the lower band of non-biotinylated form was discarded with flow-through (Figure 2C). In addition, we applied avidin-conjugated fluorophore to detect biotinylation. Imaging-based dot blot assay corroborated the RNA product being biotin-tagged in the presence of ADPRC (Figure 2D). To examine the specificity, we subjected a mixture of m^7^Gppp-RNA (45 nt) and NAD-RNA (38 nt) with 100 mM HEEB to the reaction. While NAD-RNA reacted with HEEB, as indicated by the presence of a biotinylated form, m^7^G-RNA had no such a product, reflecting specificity (Figure 3A). However, we noted that HEEB, only at high concentration (400 mM), reacted with m^7^Gppp-RNA, yielding an upper band in the gel (Figure 3B). Although we used 100 mM HEEB for the subsequent ONE-seq experiments, we worried that, biotinylated m^7^Gppp-RNA, even at low level, would cause false-positive signals.

### The efficacy of NudC

We evaluated the efficacy of NudC. Our rationale lies at the fact that NudC can cleave the pyrophosphate group of NAD-RNA, while being inactive to the m^7^G cap that comprises triphosphate moiety. As noted, NudC has been previously used by the CapZyme-seq method to study the global profile of NCIN-capped RNAs from total RNA extract (34). However, CapZyme-seq cannot specifically identify NAD-RNAs *in vivo* (34). Moreover, studies reported non-specific activity of NudC on m^7^G-RNA in the presence of Mn^2+^ and upon prolonged incubation (35,36); we therefore performed NudC without Mn^2+^ and within 30 min of treatment. We designed different spike-in RNAs to assess the performance of NudC treatment.

First, 38 nt RNA oligos with either NAD or m^7^G-cap were tested. NudC was able to de-cap NAD-RNA (38 nt) but not m^7^Gppp-RNA (38 nt) as shown by a lower-sized band corresponding to the de-capped product (Figure 3C). We further subjected a mixture of NAD-RNA (38 nt) and m^7^Gppp-RNA (45 nt) to the same reaction. The result clearly showed that NudC was able to selectively de-cap NAD-capped (38 nt) but not m^7^G-capped RNA (45 nt) (Figure 3D). We tested the ability of NudC to elute biotinylated RNAs bound by streptavidin beads. To ensure complete elution, we first used biotin to replace all bound RNAs from the streptavidin beads, followed by NudC treatment. Then we re-applied high-capacity streptavidin beads to capture biotinylated-RNAs that were resistant to NudC treatment. At this step, contaminating m^7^G-RNAs that remain biotinylated are retained on the beads, while decapped NAD-RNAs are eluted. Treatment by NudC efficiently eluted RNAs derived from NAD-capped (38 nt) but not m^7^Gppp-capped forms (38 nt) from streptavidin beads (Figure 3E and F). A control experiment corroborated that m^7^Gppp-RNA (38 nt), but not NAD-RNA (38 nt) was sensitive to yDcpS treatment, a decapping enzyme that hydrolyzes the triphosphate linkage of m^7^G-capped RNA (Supplementary Figure S2A).

Second, we generated three groups of 106 nt synthetic RNA as spike-ins (i.e. ppp-RNA without a cap, NAD-capped RNA, and m^7^G-capped RNA (m^7^Gppp-RNA), respectively). HEEB reacted with NAD-RNA (106 nt) and m^7^Gppp-RNA (106 nt), but not ppp-RNA (106 nt), resulting in a band retained by the streptavidin beads (Supplementary Figure S2B). We subjected total RNA extract mixed with spike-in RNA to ONE-seq experiment, followed by qRT-PCR. NAD-RNA (106 nt), in an ADPRC-dependent manner, were robustly and significantly enriched compared to ppp-RNA (106 nt) (Figure 3G). In the presence of ADPRC, m^7^Gppp-RNA (106 nt) could be detected on streptavidin beads; treatment of NudC, however, found no evidence to elute m^7^Gppp-RNA (106 nt) (Figure 3H).

Third, we synthesized two long RNA spike-ins with identical sequence (500 nt) but had either NAD or m^7^G-cap, followed by polyA tails. Presumably, endogenous transcripts may contain both NAD and m^7^G-capped forms, though the percentage may differ for particular genes. The rationale of this design is to mimic endogenous genes that have either low (0% or 1%) or relatively high (5% or 10%) NAD modification. Total RNAs were mixed with spike-ins that had different ratios of NAD-RNA and m^7^G-RNA. The sample that contained 100% m^7^G-RNA spike-in represented a gene with no NAD capping, which allowed the assessment of the specificity of ONE-seq platform. Other samples that contained 1%, 5%, and 10% of NAD- relative to m^7^G-RNA spike-ins were used to determine the capture sensitivity. We subjected 100 μg total RNA from mouse livers mixed with 1 ng spike-in RNA to ONE-seq experiment, followed by polyA-selected RNA sequencing. In the sample mixed with 100% m^7^G-RNA spike-in, we found no enrichment (Figure 3I). As demonstrated by sequence read count, the amount of spike-in RNA exceeded 99% of endogenous mRNA transcripts from mouse livers (Supplementary Figure 4A), representing an abundant transcript in the transcriptome. This evidence highlights the specificity of ONE-seq, which can eliminate potential contamination from m^7^G-capped transcripts that are highly expressed. In the sample that contained 1% of NAD-capped forms, the enrichment was low and variable (Figure 3I), suggesting that ONE-seq might not be sensitive enough to capture low-degree NAD modification for particular transcripts. In contrast, when NAD-capped forms accounted for 5% or 10% of the spike-in transcript, the level increased up to 3.4-fold and 4-fold, respectively (Figure 3I), reflective of significant enrichment. This experiment provided an estimate of the stoichiometry of NAD versus m^7^G in the endogenous transcripts by leveraging spike-in RNAs with ascending ratios of NAD-capped forms. As a result, we proceeded to set 2-fold enrichment, roughly reflecting 3% of NAD-capped form for a particular transcript, between ONE-seq and input as the cutoff for the identification of NAD-RNAs.

Fourth, we determined the noise-cancelling effect of NudC by comparative analysis of RNA-seq datasets. Total RNAs, after HEEB reaction, were captured by streptavidin beads, followed by either NudC-catalyzed elution (NudC+) or mock treatment (NudC-) (Supplementary Figure S3). polyA-selected RNA sequencing was performed. To pinpoint NAD-capped RNA, we set 2-fold enrichment of read counts as the cutoff (Supplementary Figure S3). From mouse liver tissues, 1,952 NAD-RNAs were identified in the absence of NudC; however, the usage of NudC expelled 489 genes from the profile (Figure 3J and Supplementary Table S1). As a result, NudC treatment may contribute by roughly reducing 25% of the false positive hits presumably derived from m^7^G-capped RNAs.

### Epitranscriptome-wide analysis of NAD-RNAs by ONE-seq

We tested the utility of ONE-seq. We profiled NAD-RNAs from mouse liver tissues of young (2-month) and aged (18-month) cohorts. After quality control, we obtained in average ∼12.3 million high-quality and uniquely mapped sequencing read pairs from each library (Supplementary Figure S4B). Assessment of datasets corroborated that sequencing saturation has been reached (Supplementary Figure S4C). Principal component analysis (PCA) illustrated that the biological replicates of each sample type clustered together, reflecting high reproducibility of the experiments (Supplementary Figure S4D). We proceeded to set 2-fold enrichment of read counts as the cutoff, which led us to identify 2017 and 1820 NAD-RNAs from young and aged animals, respectively (Supplementary Table S2). Notably, similar distributions of read counts along gene bodies were shown between mRNA transcriptome (input) and NAD epitranscriptome (ONE-seq) (Supplementary Figure S4E), suggesting comparable integrity of transcripts between m^7^G and NAD-capping.

### Validation of NAD-RNAs by boronic acid

**Figure 4.**
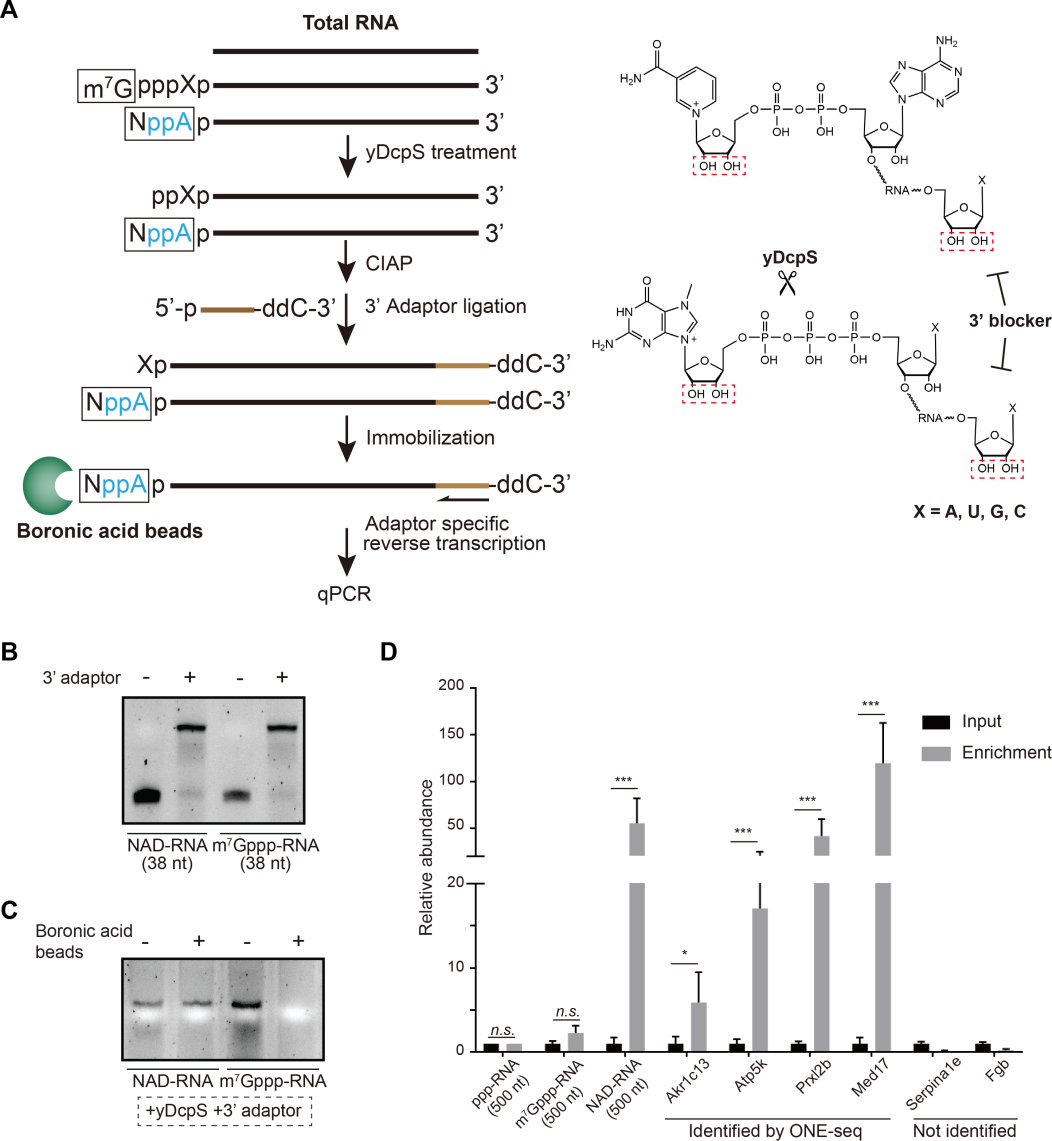
Validation of NAD-RNAs by boronate affinity, an ADPRC-independent method. (**A**) The workflow of boronic acid beads-based validation strategy (left panel). In the presence of yDcpS, 7-methylguanosine of the m^7^G-cap was removed. Prior to 3′-end ligation, RNAs were treated with CIAP to remove 5′-terminal phosphate. Adapters with 2′,3′-dideoxycytidine (ddC) were ligated for blockage of the *cis*-diols moiety at the 3′-end of RNA. At this step, affinity binding can only occur between the boronyl group from boronic acid and 1,2-cis diols from the nicotinamide riboside of NAD-cap. Adaptor specific reverse transcription, followed by gene-specific qPCR, was performed to assess the NAD modification. In the right panel, 1,2-cis diols were highlighted in red dash rectangles. (**B**) Ligation of 3′adaptor resulted in the appearance of upper bands. (**C**) NAD-RNA, but not m^7^Gppp-RNA RNA oligos, were retained by boronic acid beads, after yDcpS treatment and 3′-adaptor ligation. (**D**) Assessment of gene-specific NAD-capping by qRT-PCR. Based on boronic acid beads, Akr1c13, Atp5k, Prxl2b, and Med17 identified by ONE-seq as well as Serpinale and Fgb not identified by ONE-seq were examined. (Two-tailed Student's *t* test: ***P < 0.001, *P < 0.01; n.s., not significant).

We validated newly identified NAD-RNAs by an ADPRC-independent method. To do this, we applied boronic acid beads. Our rationale lies at the fact that boronyl groups of boronic acid can form relatively stable complexes with 1,2-*cis* diols, occurring naturally at the 3′-end of RNA, as well as in the 7-methylguanosine of m^7^G-cap and the nicotinamide riboside of NAD-cap (37–39). Inspired by previous assays (37,38), we devised a qRT-PCR strategy to specifically capture NAD-RNA (Figure 4A). First, yDcpS is used to remove 7-methylguanosine at the 5′-end of m^7^G-RNA, while nicotinamide riboside of NAD-cap remains intact. Second, an RNA adaptor that contains a 2′,3′-dideoxycytidine (ddC) at the terminal is ligated to the 3′-end of RNA, such that the vicinal-diol moiety of the ribose at the 3′-end of RNA no longer exists. At this step, affinity binding can only occur between the boronyl group from boronic acid and 1,2-cis diols from the nicotinamide riboside of NAD-cap. Importantly, we perform reverse transcription using an adaptor-specific primer to harvest RNAs with 3′end blocker, thereby abolishing false signals from boronate binding of RNAs that are not properly ligated with adaptor. Consequently, only RNA transcripts that contain NAD-cap can be selectively enriched by boronate affinity.

As shown in Supplementary Figure S2A, yDcpS treatment specifically de-capped m^7^G, but not NAD, from RNA oligos (38 nt). In the presence of RNA ligase, 3′adaptor was efficiently ligated to the spike-in RNAs (38 nt), yielding an upper band in the gel (Figure 4B). Upon these treatments, we showed the evidence that NAD-capped, but not m^7^G-capped RNA oligos, were retained by boronic acid beads (Figure 4C). We then extended this strategy to the total RNA extracts from mouse livers mixed with three long spike-in RNAs (ppp-RNA and m^7^Gppp-RNA as negative controls, NAD-RNA as positive control) that were designed with different sequences. This experiment clearly demonstrated that NAD-RNA (500 nt) spike-ins, but not ppp-RNA (500 nt) and m^7^Gppp-RNA (500 nt), were selectively and significantly enriched by boronic acid beads (Figure 4D). We proceeded to successively validate the NAD-capping for four endogenous transcripts, Akr1c13, Atp5k, Prxl2b, and Med17, as identified by ONE-seq (Figure 4D). In contrast, two genes that were not identified by ONE-seq, Serpinale and Fgb, showed no enrichment by boronic acid beads (Figure 4D). Combined, this ADPRC-independent validation corroborated newly identified NAD-RNAs, supporting the notion that ONE-seq, an ADPRC-dependent method, enables robust and efficient identification of NAD-RNAs.

### Characterization of NAD-RNAs from mouse livers

**Figure 5.**
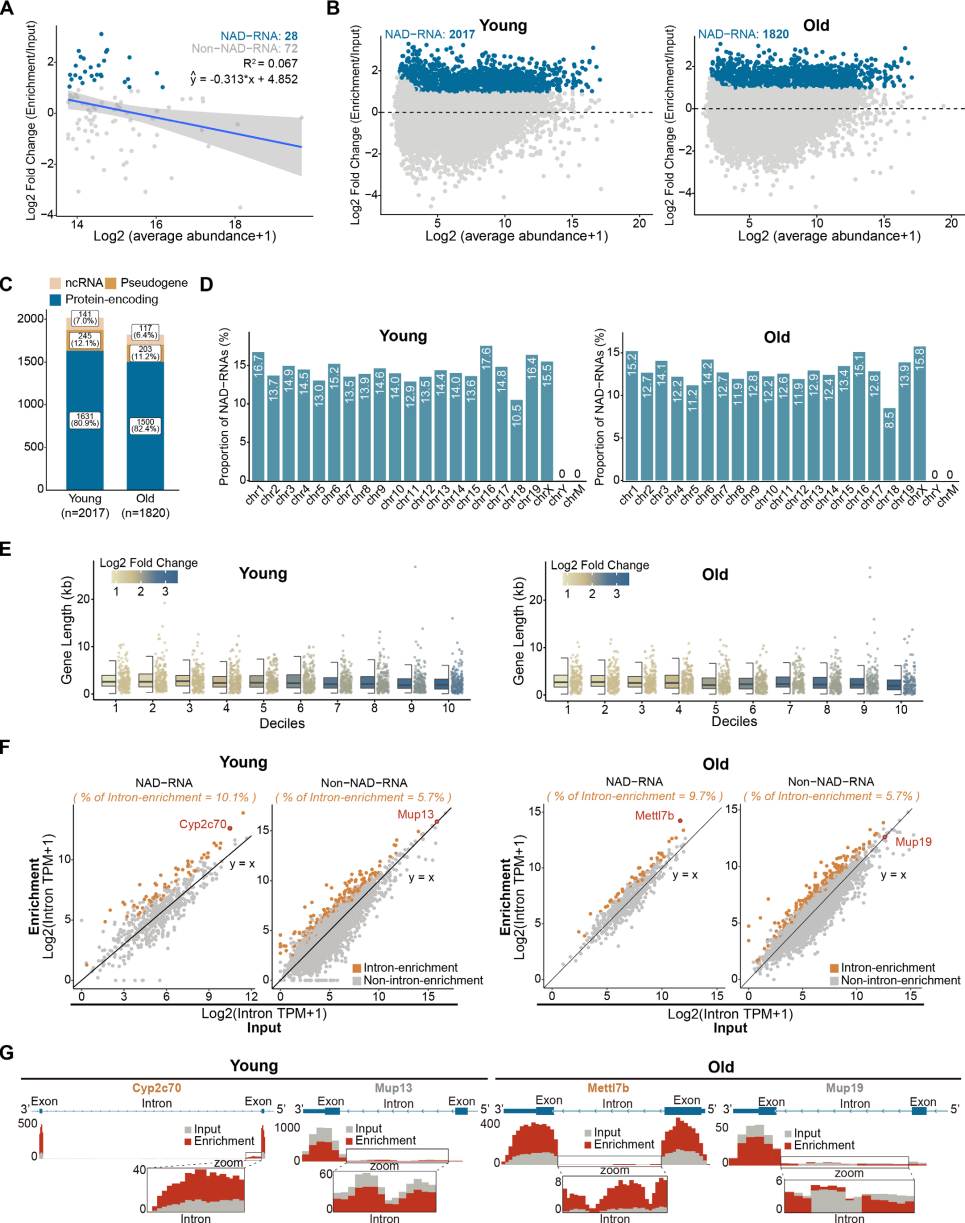
Epitranscriptome-wide profiling of NAD-RNAs by ONE-seq. (**A**) Scatter plots showing no positive correlation of the enrichment levels and transcript abundances among the top 100 abundant transcripts from livers of young mice (2-month). Blue dots represented the NAD-RNAs identified by ONE-seq. The blue line was the linear regression fits and grey shade area was the 95% confidence interval. (**B**) Scatter plots showing NAD-RNAs (blue dots) identified by ONE-seq in young (left panel) and aged (right panel) animals. Two-fold enrichment of read counts was used as the cutoff. 2017 and 1820 NAD-RNAs from young (2-month) and aged (18-month) mouse livers, respectively. Total RNAs were from mouse livers of indicated age. (**C**) NAD capping on different RNA types, with most occurrence on protein-encoding genes (blue), but also on non-coding RNAs (orange) and pseudogenes (yellow). (**D**) Chromosomal distribution shows that NAD-RNAs are derived from genes localized on autosomes and X chromosomes, but not from the Y chromosome and the mitochondrion genome. (**E**) From 10 deciles based on enrichment, genes with short length tend to have increased modification of NAD. (**F**) NAD-RNA tends to have higher ratio of intron retention than non-NAD capped forms. (**G**) Genome browser views illustrate the presence of intron read counts in select NAD-capped genes.

We characterized newly identified NAD-RNAs from mouse livers by ONE-seq. We extracted top 100 most abundant transcripts from input dataset and examined their relative enrichment. This analysis found no evidence of positive correlation, indicating that NAD capping might not be simply incidental events that are proportionally correlated with transcript abundance (Figure 5A). In mouse livers, NAD capping mostly occurred on protein-encoding genes, but also extended to non-coding RNAs and pseudogenes (Figure 5B and C). NAD-RNAs were shown to be derived from genes localized on autosomes and X chromosomes, but not from the Y chromosome and the mitochondrion genome (Figure 5D). By dividing NAD-RNAs into 10 deciles based on enrichment, we observed that shorter genes tended to have increased modification of NAD (Figure 5E). Analysis of the splicing events revealed a higher ratio of intron retention in NAD-capped than those of non-NAD-capped mRNA transcripts (Figure 5F). Genome browser views illustrated the presence of intron read counts in mRNAs capped by NAD, whereas those introns were normally spliced in non-NAD capped forms (Figure 5G).

**Figure 6.**
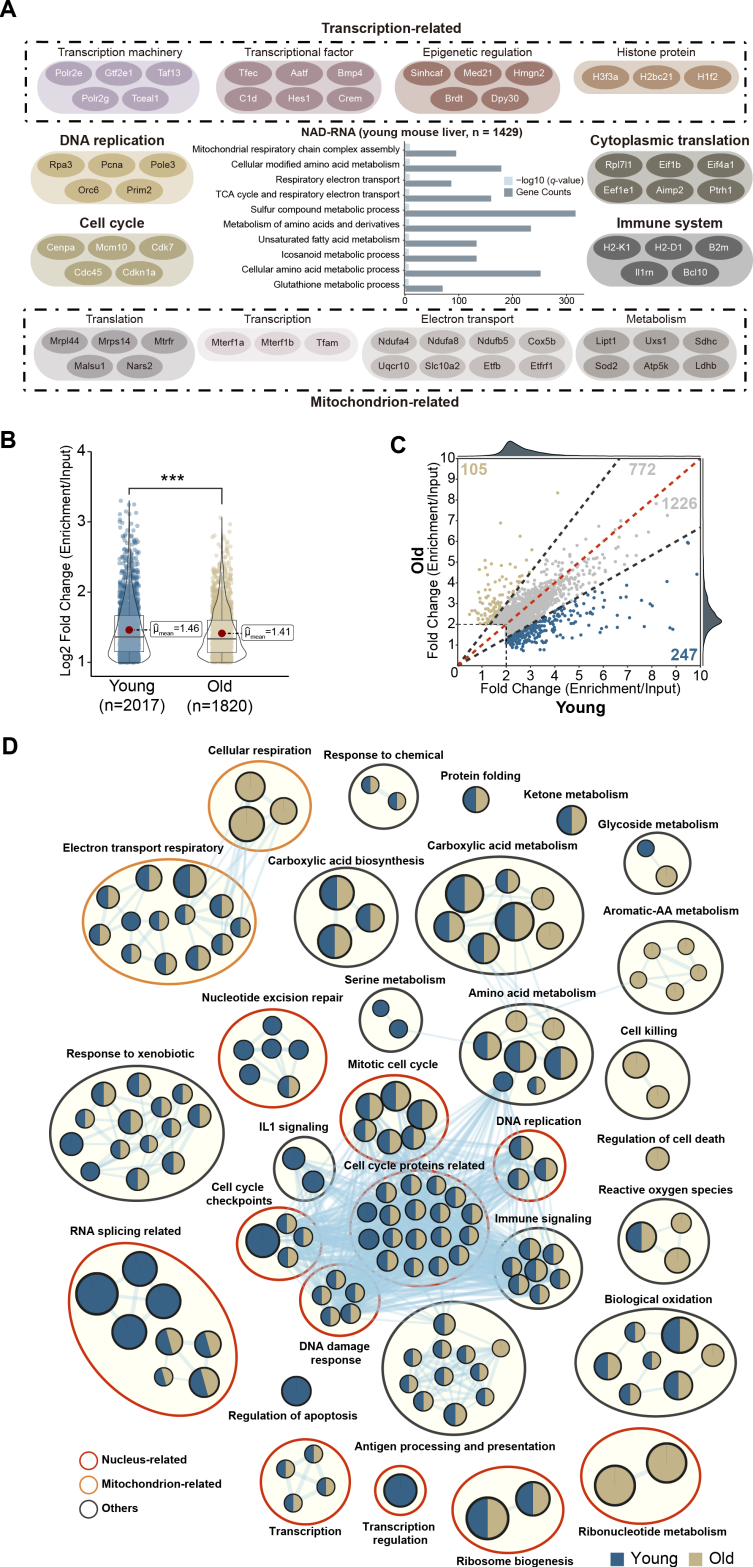
Age alters NAD-capped RNAs. (**A**) Pathway analysis reveals the biological processes of NAD-capped RNAs are mainly involved in DNA replication, transcription, cell cycle, metabolism, immune system, response to stimulus, and ribosome biogenesis. Grey dashed line in the bar plot indicates the 0.05 *q*-value cutoff. (**B**) Violin plot showing the global dynamics of NAD-RNAs during aging (***P < 0.001 by Wilcox rank-sum test). Both the number of RNAs that contain NAD-capped form and the global extent of capping become decreased in aged (18-month) compared to young (2-month) mouse livers. (**C**) Scatter plot of NAD-capped RNAs in young and aged mouse livers. Common NAD-RNAs were shown in grey. Specific NAD-RNAs were found in both young (2-month, blue) and aged (18-month, yellow) mouse livers. (**D**) Network of functional pathways for genes that contains NAD-cap from young and aged mouse livers, respectively. Nodes represent pathways and edges represent shared genes between pathways. Nodes colored in blue are young-specific pathways (2-month) and those in khaki are aged-specific pathways (18-month), while those in both colors are shared by both young and aged animals.

To gain functional insight, we performed pathways analysis on RNAs found to contain NAD-cap. We started by analyzing dataset from young mouse livers. Analysis of biological processes and molecular pathways revealed that NAD-RNAs were mainly involved in DNA replication, transcription, cell cycle, metabolism, immune system, response to stimulus, and ribosome biogenesis (Figure 6A). In addition, genes linked to subcellular localization and function, such as those in nucleus and mitochondrion, were highly enriched.

In nucleus, genes encoded basic machineries in DNA replication (e.g. DNA primase and DNA polymerase) and transcription (e.g. RNA polymerase II) were found to contain NAD-cap. Also, transcripts with 5′-NAD modification were enriched in cell cycle (e.g. centromere protein and CDK) and DNA damage responses (e.g. ATM and TP53). Not just housekeeping genes, transcriptional factors (e.g. BMP5 and Interferon), epigenetic pathways (e.g. Mediator Complex, DPY-30 and HMGN2), and histone-related genes were also subject to NAD modification (Figure 6A and Supplementary Table S3). In mitochondrion, we noted that its basic transcription (e.g. mitochondrial transcription termination factors) and translation (e.g. mitochondrial ribosomal proteins) were highlighted by NAD-RNAs. Nuclear-encoded mitochondria genes, known to play essential roles in electron transport (e.g. mitochondrial complex I and cytochrome P450) and metabolism (e.g. SDHC and LDHB), had NAD modification (Figure 6A and Supplementary Table S3). In contrast, no transcripts encoded by the mitochondrial genome were found to be linked with NAD.

### Age alters NAD-capped RNAs

In line with an age-modulated decrease of NAD (Supplementary Figure S5), both the number of RNAs that contained NAD-capped form and the global extent of capping became decreased in aged compared to young animals (Figure 6B). Gene-specific analysis conformed with this trend of age-modulated decrease in NAD capping; however, select NAD-RNAs had higher enrichment in aged than young mouse livers (Figure 6C). Interestingly, we noted different functional categories in young and aged animals. Whereas young animals displayed enrichment of pathways involved in nucleotide excision repair (e.g. excision repair protein) and RNA splicing (e.g. splicing factor and alternative splicing regulator), aged animals tended to enrich metabolic signatures, such as ribonucleotide metabolism (e.g. UPP2) and aromatic-amino acids metabolism (e.g. TDO2), as well as oxidation-related pathways (e.g. GSTs and SODs) (Figure 6D and Supplementary Table S3). Together, our study reveals new features of age-modulated NAD-RNAs from adult mouse livers.

### ONE-seq enables gene-specific analysis of NAD-RNAs

**Figure 7.**
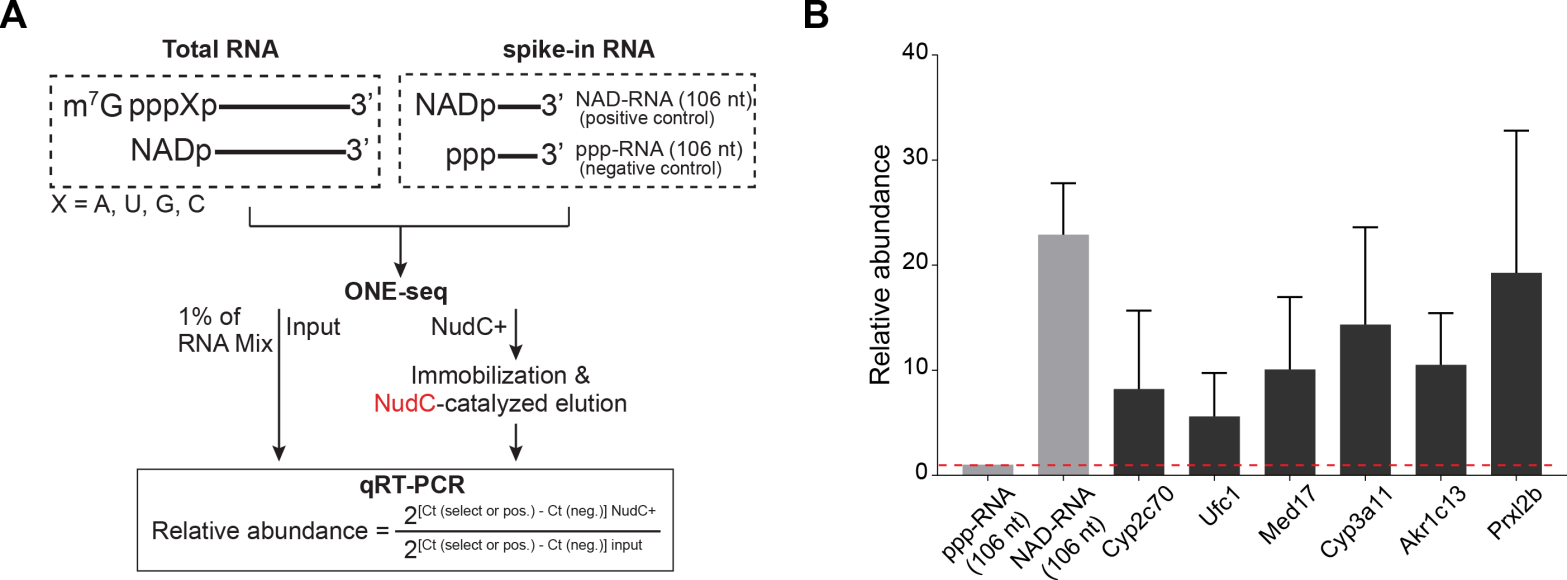
Gene-specific analysis of NAD-RNAs by ONE-seq. (**A**) Schematic workflow of gene-specific assessment of NAD modification with ONE-seq platform by qRT-PCR. ppp-RNA (106 nt) and NAD-RNA (106 nt) were included as a baseline negative control and a positive control, respectively. (**B**) Assessment of gene-specific NAD-capping by qRT-PCR. Based on ONE-seq platform, Cytochrome P450 family Cyp2c70 and Cyp3a11 involved in electron transport, Akr1c13 and Prxl2b of the metabolism-related genes, and Med17 and Ufc1 genes of gene regulatory pathways were examined. Total RNAs were from liver tissues of young mice (2-month).

Above evidence supported the notion that ONE-seq platform permits epi-transcriptome-wide profiling directly from total RNA, prompting us to extend its application for gene-specific assessment by qRT-PCR. To do this, we included non-capped ppp-RNA (106 nt) as a baseline negative control, and NAD-RNA (106 nt) as a positive control. Total RNA and internal spike-in controls were subjected to ONE-seq experiment, followed by qRT-PCR. The relative abundance was calculated between NudC + and input samples (Figure 7A). Our data demonstrated that ONE-seq enabled comparative and quantitative assessment of NAD-capping events on specific genes, such as the cytochrome P450 family Cyp2c70 and Cyp3a11 involved in electron transport, Akr1c13 and Prxl2b of the metabolism-related genes, as well as Med17 and Ufc1 genes of gene regulatory pathways (Figure 7B).

## DISCUSSION

NCIN, the nucleoside-containing metabolite such as NAD, 5′-desphospho coenzyme A (dpCoA), flavin adenine dinucleotide (FAD), uridine diphosphate glucose (UDP-Glc) and uridine diphosphate *N*-acetylglucosamine (UDP-GlcNAc), can be incorporated at the 5′-end of RNA during transcription initiation in both prokaryotes and eukaryotes (5,34). CapZyme-seq has been applied to detect the global landscape of NCIN-capped RNAs *in vivo* but this method does not pinpoint the nature NCIN cap (34). As a result, CapZyme-seq cannot be used to profile NAD-RNAs from total RNA extract.

NAD is the hub metabolite and redox regent for cells, involving in a wide range of biological processes (9). The attachment of NAD to RNA inherently connects essential metabolic regulation with gene expression, defining a critical layer of epi-transcriptomic regulation. However, investigating biological insight of NAD-RNAs has been hindered by the analytical methods available. The currently reported NAD-RNA identification methods involve the use of multiple reactions, and each reaction requires additional steps of RNA clean-up and precipitation. In addition, the canonical 5′-end cap structure of RNA (m^7^G) has been found to contaminate the NAD-RNA profile. As such, SPAAC-NAD-seq (16), the most recent method developed in *Arabidopsis*, introduces antibody-based pre-treatment to deplete m^7^G-RNA from purified mRNA. Consequently, these methods demand laborious procedures and high RNA input, which cannot be readily applied for gene-specific analysis. In the current study, we design a one-step chemo-enzymatic reaction by HEEB that directly conjugates NAD-RNA with biotin affinity tag. We apply NudC-based post-treatment to specifically harvest NAD-capped RNAs, allowing the assay to be simply performed from total RNA. Compared to previous methods, ONE-seq requires significantly less amount of RNA input. More importantly, we include different types of spike-in RNAs to carefully demonstrate that ONE-seq can capture NAD-RNAs with specificity and sensitivity, especially for those that have equal to or higher than 3% of NAD-capped form for a particular transcript (see Figure 3I). However, one limitation to note is that ONE-seq is not sensitive enough to detect low-degree NAD modification, such as those equal to or lower than 1% of the transcript (see Figure 3I). For future experiments, synthetic m^7^G- and NAD-RNA spike-ins can be included as quality controls to determine specificity and sensitivity, respectively. Inspired by previous work (38,39), we devise an ADPRC-independent assay by boronate affinity to validate NAD-RNAs. By circumventing the need for boronic acid gel electrophoresis and radioactive probe labeling, our current assay that adopts qRT-PCR allows quantitative analysis of the capping event for multiple genes, which can be readily extended into epitranscriptomic application. Our validation supports the notion that ONE-seq is a reliable method for the identification of NAD-capped RNAs.

ONE-seq accelerates the discovery of new NAD-RNAs. NAD-capping may define a conserved gene regulatory mechanism. Consistent with the observation in *Arabidopsis* (40), NAD capping in mouse tends to occur on genes with shorter length. However, NAD, as a non-canonical initiating nucleotide, can be added to the 5′-end of RNA during transcription initiation by RNA Polymerase II. It remains enigmatic how RNA Polymerase II is able to distinguish the length of genes at the step of transcription initiation. Future investigations, including in-depth comparative analysis of NAD-RNA profiles from different organisms, may provide important information. Similar to yeast (41), transcripts with NAD-cap are more likely to have intron retention than those capped by m^7^G. In addition, we reveal prominent features of NAD-RNAs in adult mouse livers. Majority of NAD-RNAs are produced by protein-encoding genes, with their biological functions mainly involved in basic cellular events, such as DNA replication, transcription, translation, and metabolism. It is interesting to note that, although few NAD-RNAs are produced from mitochondrial-encoded genes, we determine that more than 289 nucleus-encoded genes known to function in mitochondria contain NAD capped forms. Despite these observations, however, whether NAD-RNAs would function similarly or differently from their canonical m^7^G-capped counterparts remains elusive. Thus far, the only proposed effect of NAD modification on RNAs is to trigger RNA degradation (42). However, since NAD is a well-characterized redox reagent and a widely used protein ligand, it is tempting to speculate that NAD capping may endow the expressed RNA transcripts with additional interaction, structural or regulatory properties.

ONE-seq reveals the dynamics of NAD-RNAs during life-course. Seminal studies have heightened the fact that the cellular level of NAD decreases with age, predisposing individuals to physiological decline as well as late-onset diseases (43). Our quantitative assessment describes that aging couples with a decline of NAD modification on RNAs. This data indicates that the capping event at the transcriptome level might be intimately connected to the cellular reserve of NAD. Intriguingly, despite the fact that age couples with a decline in NAD, unique NAD-RNAs can be found in aged animals, suggesting that NAD modification on specific RNAs may also be fine-tuned by the physiological status of cells. Based on the same platform, qRT-PCR analysis on specific NAD-RNAs can be applied, which potentially can be developed as biomarkers for health and disease. Aging is a complex process that could be regulated by multiple mechanisms; therefore, it would be interesting to test whether aging might be through at least in part by the alteration of NAD-capped RNAs.

Taken together, we propose ONE-seq as a simple, specific, and versatile method for the investigation of NAD-capped RNAs in a wide-spectrum of biological contexts. NAD-functionalized RNAs, poised to integrate metabolome and transcriptome, prompt future investigation into how this epi-transcriptomic mechanism may impact the physiological and perhaps pathological processes.

## DATA AVAILABILITY

All high-throughput RNA sequencing data as well as transcript quantifications have been deposited at the Gene Expression Omnibus under accession number GSE194271. Genome browser views for Cyp2c70 (http://genome.ucsc.edu/s/lda97/Cyp2c70_ONE-seq), Mup13 (http://genome.ucsc.edu/s/lda97/Mup13_ONE-seq), Mup19 (http://genome.ucsc.edu/s/lda97/Mup19_ONE-seq) and Mettl7b (http://genome.ucsc.edu/s/lda97/Mettl7b_ONE-seq) can be accessed at UCSC Genome Browser online sessions. All source codes for data analysis are available at Zenodo https://doi.org/10.5281/zenodo.7293917.

## Supplementary Material

gkac1136_Supplemental_Files

## References

[1] Topisirovic I., Svitkin Y.V., Sonenberg N., Shatkin A.J. Cap and cap-binding proteins in the control of gene expression. Wiley Interdiscip. Rev. RNA. 2011; 2:277–298. 21957010 10.1002/wrna.52

[2] Ramanathan A., Robb G.B., Chan S.-H. mRNA capping: biological functions and applications. Nucleic Acids Res. 2016; 44:7511–7526. 27317694 10.1093/nar/gkw551 PMC5027499

[3] Galloway A., Cowling V.H. mRNA cap regulation in mammalian cell function and fate. Biochim. Biophys. Acta Gene Regul. Mech. 2019; 1862:270–279. 30312682 10.1016/j.bbagrm.2018.09.011 PMC6414751

[4] Chen Y.G., Kowtoniuk W.E., Agarwal I., Shen Y., Liu D.R. LC/MS analysis of cellular RNA reveals NAD-linked RNA. Nat. Chem. Biol. 2009; 5:879–881. 19820715 10.1038/nchembio.235 PMC2842606

[5] Wang J., Alvin Chew B.L., Lai Y., Dong H., Xu L., Balamkundu S., Cai W.M., Cui L., Liu C.F., Fu X.Y. et al. Quantifying the RNA cap epitranscriptome reveals novel caps in cellular and viral RNA. Nucleic Acids Res. 2019; 47:e130. 31504804 10.1093/nar/gkz751 PMC6847653

[6] Lopez-Otin C., Blasco M.A., Partridge L., Serrano M., Kroemer G. The hallmarks of aging. Cell. 2013; 153:1194–1217. 23746838 10.1016/j.cell.2013.05.039 PMC3836174

[7] Lopez-Otin C., Galluzzi L., Freije J.M.P., Madeo F., Kroemer G. Metabolic control of longevity. Cell. 2016; 166:802–821. 27518560 10.1016/j.cell.2016.07.031

[8] Ma Z., Wang H., Cai Y., Wang H., Niu K., Wu X., Ma H., Yang Y., Tong W., Liu F. et al. Epigenetic drift of H3K27me3 in aging links glycolysis to healthy longevity in drosophila. Elife. 2018; 7:e35368. 29809154 10.7554/eLife.35368 PMC5991832

[9] Katsyuba E., Romani M., Hofer D., Auwerx J. NAD(+) homeostasis in health and disease. Nat. Metab. 2020; 2:9–31. 32694684 10.1038/s42255-019-0161-5

[10] Yoshino J., Mills K.F., Yoon M.J., Imai S. Nicotinamide mononucleotide, a key NAD(+) intermediate, treats the pathophysiology of diet- and age-induced diabetes in mice. Cell Metab. 2011; 14:528–536. 21982712 10.1016/j.cmet.2011.08.014 PMC3204926

[11] Anderson R.M., Bitterman K.J., Wood J.G., Medvedik O., Cohen H., Lin S.S., Manchester J.K., Gordon J.I., Sinclair D.A. Manipulation of a nuclear NAD+ salvage pathway delays aging without altering steady-state NAD+ levels. J. Biol. Chem. 2002; 277:18881–18890. 11884393 10.1074/jbc.M111773200

[12] Mouchiroud L., Houtkooper R.H., Moullan N., Katsyuba E., Ryu D., Cantó C., Mottis A., Jo Y.S., Viswanathan M., Schoonjans K. et al. The NAD(+)/Sirtuin pathway modulates longevity through activation of mitochondrial UPR and FOXO signaling. Cell. 2013; 154:430–441. 23870130 10.1016/j.cell.2013.06.016 PMC3753670

[13] Balan V., Miller G.S., Kaplun L., Balan K., Chong Z.Z., Li F., Kaplun A., VanBerkum M.F.A., Arking R., Freeman D.C. et al. Life span extension and neuronal cell protection by drosophila nicotinamidase. J. Biol. Chem. 2008; 283:27810–27819. 18678867 10.1074/jbc.M804681200 PMC2562057

[14] Zhang H., Ryu D., Wu Y., Gariani K., Wang X., Luan P., D’Amico D., Ropelle E.R., Lutolf M.P., Aebersold R. et al. NAD⁺ repletion improves mitochondrial and stem cell function and enhances life span in mice. Science. 2016; 352:1436–1443. 27127236 10.1126/science.aaf2693

[15] Cahova H., Winz M.L., Hofer K., Nubel G., Jaschke A. NAD captureSeq indicates NAD as a bacterial cap for a subset of regulatory RNAs. Nature. 2015; 519:374–377. 25533955 10.1038/nature14020

[16] Hu H., Flynn N., Zhang H., You C., Hang R., Wang X., Zhong H., Chan Z., Xia Y., Chen X. SPAAC-NAD-seq, a sensitive and accurate method to profile NAD(+)-capped transcripts. Proc. Natl. Acad. Sci. U.S.A. 2021; 118:e2025595118. 33753511 10.1073/pnas.2025595118 PMC8020637

[17] Winz M.L., Cahová H., Nübel G., Frindert J., Höfer K., Jäschke A. Capture and sequencing of NAD-capped RNA sequences with NAD captureSeq. Nat. Protoc. 2017; 12:122–149. 27977022 10.1038/nprot.2016.163

[18] Shao X., Zhang H., Yang Z., Zhong H., Xia Y., Cai Z. NAD tagSeq for transcriptome-wide identification and characterization of NAD(+)-capped RNAs. Nat. Protoc. 2020; 15:2813–2836. 32747820 10.1038/s41596-020-0363-z

[19] Sheng W., LaFleur M.W., Nguyen T.H., Chen S., Chakravarthy A., Conway J.R., Li Y., Chen H., Yang H., Hsu P.H. et al. LSD1 ablation stimulates Anti-tumor immunity and enables checkpoint blockade. Cell. 2018; 174:549–563. 29937226 10.1016/j.cell.2018.05.052 PMC6063761

[20] Krueger F. Trim galore. A Wrapper Tool Around Cutadapt and FastQC to Consistently Apply Quality and Adapter Trimming to FastQ Files. 2015; 516:517.

[21] Dobin A., Davis C.A., Schlesinger F., Drenkow J., Zaleski C., Jha S., Batut P., Chaisson M., Gingeras T.R. STAR: ultrafast universal RNA-seq aligner. Bioinformatics. 2013; 29:15–21. 23104886 10.1093/bioinformatics/bts635 PMC3530905

[22] Liao Y., Smyth G.K., Shi W. featureCounts: an efficient general purpose program for assigning sequence reads to genomic features. Bioinformatics. 2014; 30:923–930. 24227677 10.1093/bioinformatics/btt656

[23] Lee S., Zhang A.Y., Su S., Ng A.P., Holik A.Z., Asselin-Labat M.L., Ritchie M.E., Law C.W. Covering all your bases: incorporating intron signal from RNA-seq data. NAR Genom. Bioinform. 2020; 2:lqaa073. 33575621 10.1093/nargab/lqaa073 PMC7671406

[24] Ramírez F., Ryan D.P., Grüning B., Bhardwaj V., Kilpert F., Richter A.S., Heyne S., Dündar F., Manke T. deepTools2: a next generation web server for deep-sequencing data analysis. Nucleic Acids Res. 2016; 44:W160–W165. 27079975 10.1093/nar/gkw257 PMC4987876

[25] Thorvaldsdóttir H., Robinson J.T., Mesirov J.P. Integrative genomics viewer (IGV): high-performance genomics data visualization and exploration. Brief. Bioinform. 2013; 14:178–192. 22517427 10.1093/bib/bbs017 PMC3603213

[26] Wang L., Wang S., Li W. RSeQC: quality control of RNA-seq experiments. Bioinformatics. 2012; 28:2184–2185. 22743226 10.1093/bioinformatics/bts356

[27] Love M.I., Huber W., Anders S. Moderated estimation of fold change and dispersion for RNA-seq data with DESeq2. Genome Biol. 2014; 15:550. 25516281 10.1186/s13059-014-0550-8 PMC4302049

[28] Wickham H. ggplot2. Wiley Interdiscip. Rev. Comput. Stat. 2011; 3:180–185.

[29] Kolberg L., Raudvere U., Kuzmin I., Vilo J., Peterson H. gprofiler2 – an R package for gene list functional enrichment analysis and namespace conversion toolset g:Profiler. F1000Res. 2020; 9:ELIXIR–709. 10.12688/f1000research.24956.1 PMC7859841 33564394

[30] Jassal B., Matthews L., Viteri G., Gong C., Lorente P., Fabregat A., Sidiropoulos K., Cook J., Gillespie M., Haw R. et al. The reactome pathway knowledgebase. Nucleic Acids Res. 2020; 48:D498–D503. 31691815 10.1093/nar/gkz1031 PMC7145712

[31] Gene Ontology Consortium The gene ontology resource: enriching a GOld mine. Nucleic Acids Res. 2021; 49:D325–D334. 33290552 10.1093/nar/gkaa1113 PMC7779012

[32] Shannon P., Markiel A., Ozier O., Baliga N.S., Wang J.T., Ramage D., Amin N., Schwikowski B., Ideker T. Cytoscape: a software environment for integrated models of biomolecular interaction networks. Genome Res. 2003; 13:2498–2504. 14597658 10.1101/gr.1239303 PMC403769

[33] Reimand J., Isserlin R., Voisin V., Kucera M., Tannus-Lopes C., Rostamianfar A., Wadi L., Meyer M., Wong J., Xu C. et al. Pathway enrichment analysis and visualization of omics data using g:Profiler, GSEA, cytoscape and enrichmentmap. Nat. Protoc. 2019; 14:482–517. 30664679 10.1038/s41596-018-0103-9 PMC6607905

[34] Vvedenskaya I.O., Bird J.G., Zhang Y., Zhang Y., Jiao X., Barvik I., Krasny L., Kiledjian M., Taylor D.M., Ebright R.H. et al. CapZyme-Seq comprehensively defines promoter-sequence determinants for RNA 5' capping with NAD(+). Mol. Cell. 2018; 70:553–564. 29681497 10.1016/j.molcel.2018.03.014 PMC5935523

[35] Grudzien-Nogalska E., Wu Y., Jiao X., Cui H., Mateyak M.K., Hart R.P., Tong L., Kiledjian M. Structural and mechanistic basis of mammalian nudt12 RNA deNADding. Nat. Chem. Biol. 2019; 15:575–582. 31101919 10.1038/s41589-019-0293-7 PMC6527130

[36] Ray A., Frick D.N. Fluorescent probe displacement assays reveal unique nucleic acid binding properties of human nudix enzymes. Anal. Biochem. 2020; 595:113622. 32059949 10.1016/j.ab.2020.113622 PMC7087442

[37] Igloi G.L., Kössel H. Affinity electrophoresis for monitoring terminal phosphorylation and the presence of queuosine in RNA. Application of polyacrylamide containing a covalently bound boronic acid. Nucleic Acids Res. 1985; 13:6881–6898. 2414733 10.1093/nar/13.19.6881 PMC322011

[38] Nubel G., Sorgenfrei F.A., Jaschke A. Boronate affinity electrophoresis for the purification and analysis of cofactor-modified RNAs. Methods. 2017; 117:14–20. 27645507 10.1016/j.ymeth.2016.09.008

[39] Bird J.G., Basu U., Kuster D., Ramachandran A., Grudzien-Nogalska E., Towheed A., Wallace D.C., Kiledjian M., Temiakov D., Patel S.S. et al. Highly efficient 5' capping of mitochondrial RNA with NAD(+) and NADH by yeast and human mitochondrial RNA polymerase. Elife. 2018; 7:e42179. 30526856 10.7554/eLife.42179 PMC6298784

[40] Wang Y., Li S., Zhao Y., You C., Le B., Gong Z., Mo B., Xia Y., Chen X. NAD(+)-capped RNAs are widespread in the arabidopsis transcriptome and can probably be translated. Proc. Natl. Acad. Sci. U.S.A. 2019; 116:12094–12102. 31142655 10.1073/pnas.1903682116 PMC6575598

[41] Walters R.W., Matheny T., Mizoue L.S., Rao B.S., Muhlrad D., Parker R. Identification of NAD+ capped mRNAs in saccharomyces cerevisiae. Proc. Natl. Acad. Sci. U.S.A. 2017; 114:480–485. 28031484 10.1073/pnas.1619369114 PMC5255579

[42] Jiao X., Doamekpor S.K., Bird J.G., Nickels B.E., Tong L., Hart R.P., Kiledjian M. 5' End nicotinamide adenine dinucleotide cap in human cells promotes RNA decay through DXO-Mediated deNADding. Cell. 2017; 168:1015–1027. 28283058 10.1016/j.cell.2017.02.019 PMC5371429

[43] Covarrubias A.J., Perrone R., Grozio A., Verdin E. NAD(+) metabolism and its roles in cellular processes during ageing. Nat. Rev. Mol. Cell Biol. 2021; 22:119–141. 33353981 10.1038/s41580-020-00313-x PMC7963035

