# Supplementary material for: ONE-seq: epitranscriptome and gene-specific profiling of NAD-capped RNA
Source: Nucleic Acids Res. 2022 Dec 7;51(2):e12. doi: 10.1093/nar/gkac1136 (PMC9881147; doi:10.1093/nar/gkac1136)
Supplement: gkac1136_Supplemental_Files [file gkac1136_supplemental_files.zip › Supplementary data.pdf]

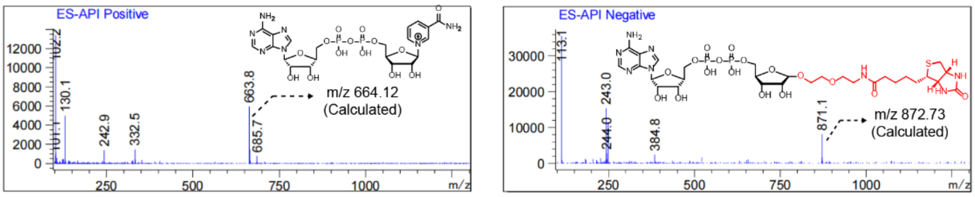

Niu et al. Supplementary Figure 1

Supplementary Figure 1: LC-MS spectra of NAD and the reaction product.

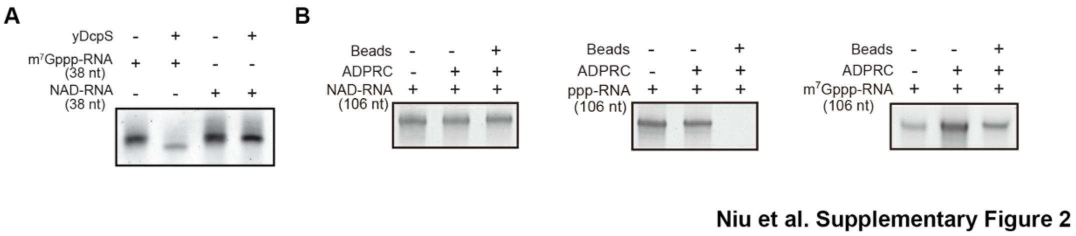

Supplementary Figure 2: Treatment of yDcpS and reaction between HEEB and different RNA capped forms. (A) yDcpS can de-cap m^{7}Gppp-RNA (38 nt), but not NAD-RNA (38 nt). (B) NAD-RNA (106 nt) and m^{7}Gppp-RNA (106 nt), but not ppp-RNA (106 nt), can be biotinylated by HEEB reaction.

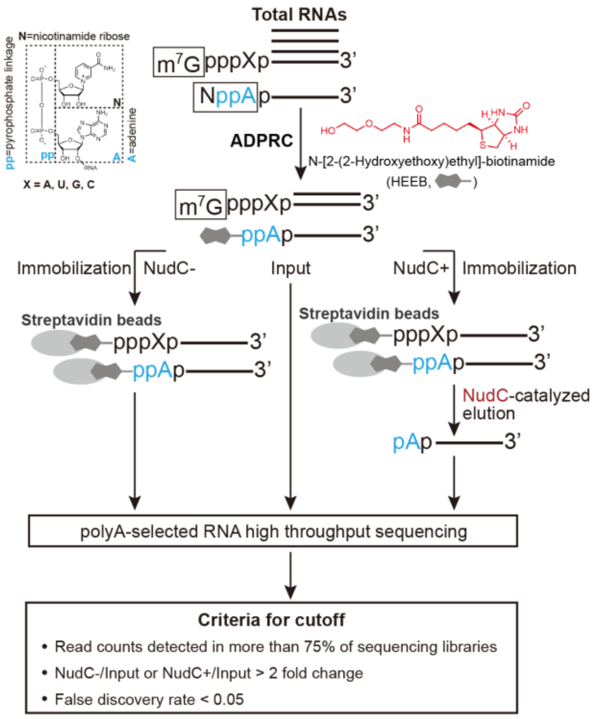

Niu et al. Supplementary Figure 3

Supplementary Figure 3: The overview to test noise-cancelling effect of NudC.

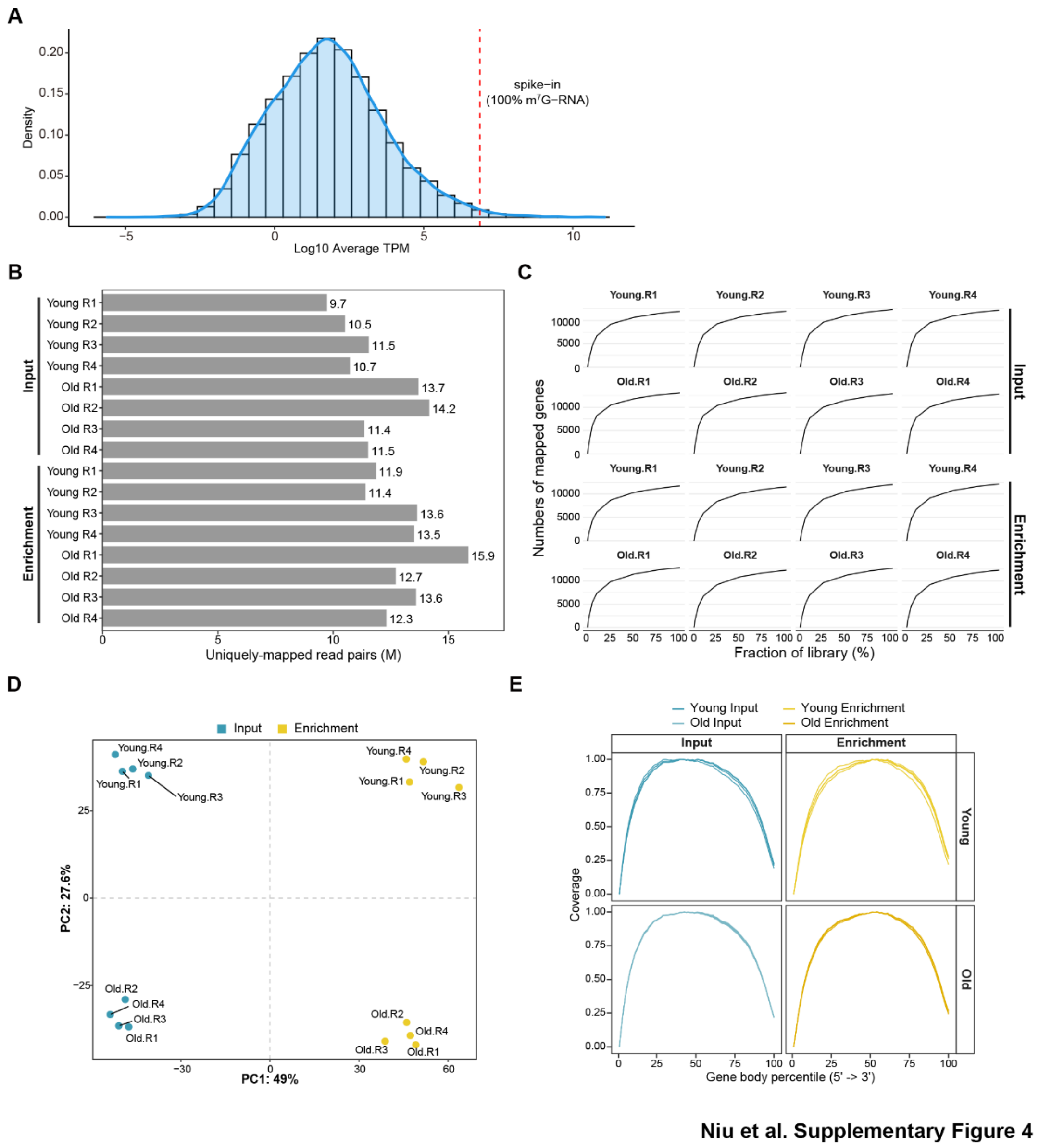

Supplementary Figure 4: Quality control of ONE-seq. (A) Histogram showing the distribution of Log10-TPM (Transcript Per Million) normalized gene expression levels from ONE-seq. Vertical dashed red line indicated the normalized transcript abundances of spike-in RNA with 100% m^7G-capped forms. (B) Analysis of sequences alignment from young (2-month) and aged (18-month) mouse livers. (C) Analysis of sequencing saturation. RNA samples as in (B). (D) Analysis of PCA. (E) Analysis of transcripts integrity. RNA samples as in (B).

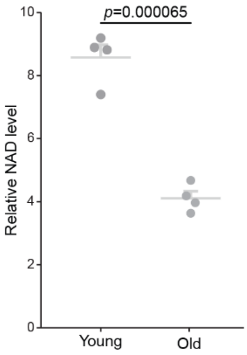

Niu et al. Supplementary Figure 5

Supplementary Figure 5: NAD decreases with age in mouse livers. Metabolites were extracted from young (2-month) and aged (18-month) mouse livers. Significance was assessed by Student's *t* test.

Supplementary Table 1. Gene lists demonstrate noise canceling effect of NudC to reduce potential false signals from m^7G-capped RNAs.

Supplementary Table 2. Gene lists of newly-identified NAD-capped RNAs by ONE-seq from liver tissues of young (2-month) and aged (18-month) mice.

Supplementary Table 3. Detailed gene and pathway analysis of NAD-capped RNAs from liver tissues of young (2-month) mice.

Supplementary Table 4. Primers used for qRT-PCR experiments.

Supplementary Table 5. RNA oligo adaptor and adapter-based primer for reverse transcription used for boronate affinity assay.
